# New Cytotoxic Terpenoids from Soft Corals *Nephthea* *chabroli* and *Paralemnalia thyrsoides*

**DOI:** 10.3390/md15120392

**Published:** 2017-12-19

**Authors:** Yu-Sheng Lee, Tsai-Hui Duh, Shu-Sheng Siao, Rey-Chang Chang, Shang-Kwei Wang, Chang-Yih Duh

**Affiliations:** 1 Department of Marine Biotechnology and Resources, National Sun Yat-sen University, Kaohsiung 804, Taiwan; m995020028@student.nsysu.edu.tw (Y.-S.L.); m035020008@student.nsysu.edu.tw (S.-S.S.); 2 Department of Medicinal and Applied Chemistry, Kaohsiung Medical University, Kaohsiung 807, Taiwan; tshudu@kmu.edu.tw; 3 Department of Marine Biotechnology, National Kaohsiung Marine University, Kaohsiung 811, Taiwan; rcchang@mail.nkmu.edu.tw; 4 Department of Microbiology, Kaohsiung Medical University, Kaohsiung 807, Taiwan

**Keywords:** *Nephthea chabroli*, *Paralemnalia thyrsoides*, cytotoxicity

## Abstract

A novel cytotoxic diterpenoid, chabrolin A (**1**) (possessing an unprecedented terpenoid skeleton), as well as three new cytotoxic sesquiterpenoids, parathyrsoidins E–G (**2**–**4**), were isolated by cytotoxicity-guided fractionation from soft corals *Nephthea* *chabroli* and *Paralemnalia thyrsoides*. The structures of the new compounds were determined by extensive analysis of spectroscopic data.

## 1. Introduction

**Figure 1:**
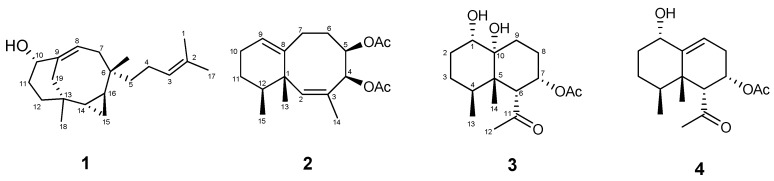
Structures of Metabolites **1**–**4**.

Soft corals belonging to the genera *Nephthea* and *Paralemnalia* have proven to be a rich source of bioactive terpenoids [1,2,3,4,5,6,7,8,9,10,11,12,13,14,15,16,17,18,19]. In searching for bioactive compounds from marine organisms, the crude extracts of soft corals *Nephthea chabroli* and *Paralemnalia thyrsoides* have been found to exhibit cytotoxicity against murine lymphcytic leukemis cell line, with IC_50_ values of 12.0 and 15.2 μg/mL, respectively. Cytotocixity-guided fractionation of soft corals *N. chabroli* and *P. thyrsoides* led to the isolation and characterization of a novel cytotoxic diterpenoid, chabrolin A (**1**) (possessing an unprecedented terpenoid skeleton), as well as three new cytotoxic sesquiterpenoids, parathyrsoidins E–G (**2**–**4**) (Figure 1). The structures of these metabolites were established by extensive spectroscopic analysis.

## 2. Results and Discussion

**Figure 2:**
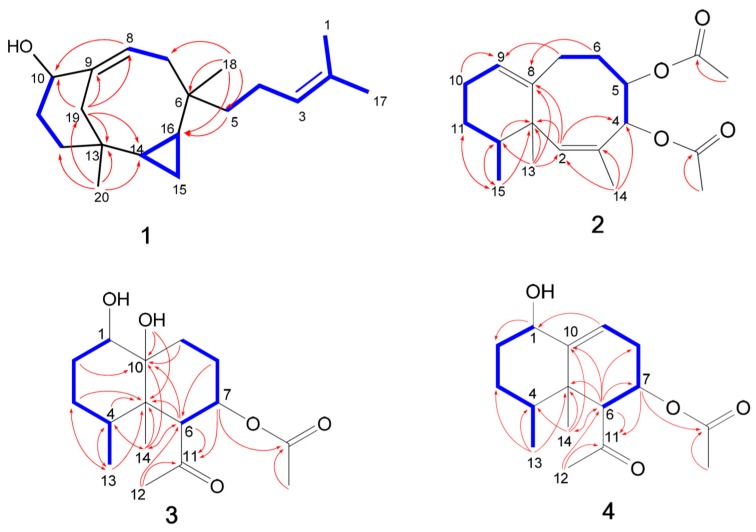
Selected ^1^H-^1^H COSY (blue bold lines) and HMBC (red arrows) correlations of **1**–**4**.

**Figure 3:**
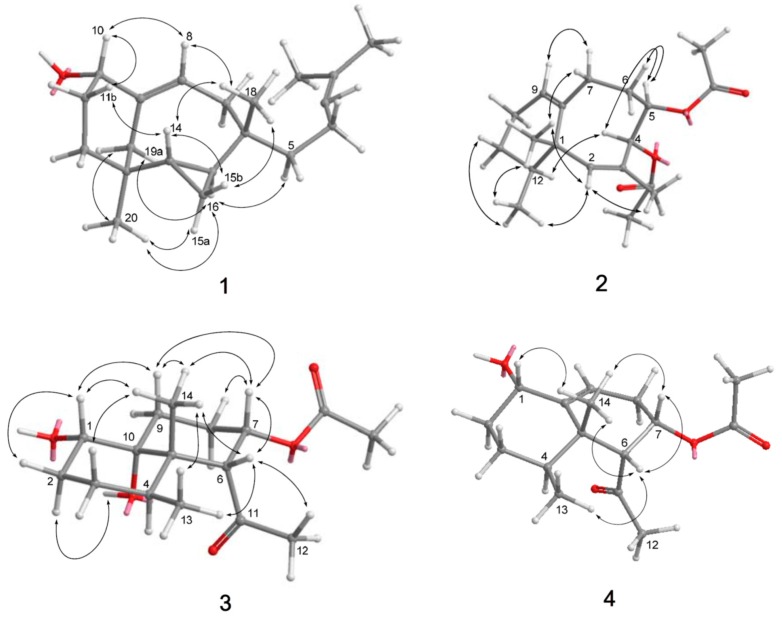
Key NOESY correlations for **1**–**4**.

The molecular formula of chabrolin A (**1**) was determined as C_20_H_32_O from its HR-FAB-MS, ^13^C NMR, and DEPT spectroscopic data. The ^13^C NMR (Figure S2) and DEPT spectrum of **1** exhibited the presence of four methyl, seven sp^3^ methylene, three sp^3^ methine, two sp^2^ methine, two sp^3^ quaternary, and two sp^2^ quaternary carbons. The presence of two trisubstituted olefins in **1** was shown by the NMR data [δ_H_ 5.59 t (*J* = 8.0 Hz), 5.13 t (*J* = 7.2 Hz); δ_C_ 144.5 C, 121.6 CH, 130.9 C, 125.3 CH] (Table 1). The NMR data [δ_H_ 0.25 dd (*J* = 15.2, 7.2 Hz), 0.36 m, 0.49 m, 0.78 dd (*J* = 14.8, 7.2 Hz); δ_C_ 8.6 CH_2_, 15.6 CH, 23.7 CH] (Table 1) pointed to a 1,2-disubstituted cyclopropane ring in **1**. The ^1^H NMR spectrum (Figure S1) displayed signals for four tertiary methyl groups, (δ_H_ 0.52, 0.64, 1.62, 1.69). The presence of the oxygen bearing sp^3^ methine (δ_C_ 74.1 CH) was shown in the ^13^C NMR spectrum. By interpretation of ^1^H-^1^H COSY correlations (Figure 2 and Figure S3), it was possible to establish four partial structures of contiguous proton systems extending from H-1 to H-5, from H-7 to H-8, from H-10 to H-12, and from H-14 to H-16. HMBC correlations (Figure S5) of (a) CH_3_-18 to C-5, C-6, C-7, and C-16, (b) H_2_-19 to C-8, C-9, C-10, C-13, and C-14, (c) H-8 to C-10, (d) CH_3_-20 to C-12, C-13, C-14, and C-19 connected four partial structures concluding the planar structure of **1**, as shown in Figure 2. The above functionalities revealed that chabrolin A (**1**) possesses a novel diterpene tricyclic skeleton. The relative configuration of all chiral centers in **1** was deduced from a NOESY experiment (Figure S6). Assuming the β-orientation of H_3_-18, NOESY correlations of H_3_-18/H-15b, H_3_-18/H-8, H-8/H-10, H-10/H-11b, H-11b/H-14, and H-14/H-15b suggested all to be on the β face of the molecule. NOESY correlations of H-15a/H-16, H-16/H-19a, H-15a/H_3_-20, H-16/H_3_-20, and H_2_-19/H_3_-20 suggested H-15a, H-16, H_2_-19, and H_3_-20 were on the α face of the molecule (Figure 3).

HRESIMS of parathyrsoidin E (**2**) exhibited a pseudomolecular ion peak at *m*/*z* 343.1887 [M + Na]^+^, consistent with the molecular formula C_19_H_28_O_4_, and six degrees of unsaturation. The IR spectrum of **2** revealed the presence of ester carbonyl group (ν_max_ 1743 cm^−1^). The ^13^C NMR spectrum of **2** (Table 2 and Figure S8) displayed 19 carbon signals, and a DEPT experiments confirmed the presence of five methyl, four sp^3^ methylene, three sp^3^ methine, two sp^2^ methine, one sp^3^ quaternary, two sp^2^ quaternary, and two carbonyl carbons. The ^13^C and ^1^H NMR spectra (Figure S7) revealed the presence of two acetoxy groups [δ_H_ 2.03 (s), δ_C_ 20.8 (CH_3_), and 170.1 (CO); δ_H_ 2.06 (s), δ_C_ 21.2 (CH_3_), and 170.3 (CO)], two trisubstituted double bonds [δ_H_ 5.29 (brs), δ_C_ 137.9 (CH), and 128.0 (C); δ_H_ 5.57 (dd, *J* = 3.6, 3.2 Hz), δ_C_ 125.9 (CH), and 141.0 (C)], and two oxygen-bearing methines [δ_H_ 5.18 (ddd, *J* = 9.2, 4.8, 3.2 Hz), δ_C_ 74.7 (CH); δ_H_ 6.63 (d, *J* = 3.2 Hz), δ_C_ 71.0 (CH)]. Thus, the bicyclic structure of **2** was revealed. From the ^1^H-^1^H COSY spectrum (Figure S9) of **2**, it was also possible to identify two different structural units (Figure 2), which were assembled with the assistance of HMBC experiments (Figure S11). Key HMBC correlations (Figure 2) from H-2 to C-1, C-4, C-8, C-13, and C-14, H_2_-6 to C-4, C-5, and C-8, H_2_-11 to C-1, C-9, C-10, and C-12, H_3_-13 to C-1, C-2, C-8, and C-12, H_3_-14 to C-2, C-3, and C-4, and H_3_-15 to C-1, C-11, and C-12 permitted the connection of the carbon skeleton. Thus, **2** was identified as a neolemnane-type new compound, on the basis of the above analysis and NMR data comparison with its stereoisomers, paralemnolins E and [9].

The *Z* geometry of the 2,3-double bond was established by a NOESY correlation (Figure S12) between H-2 and H_3_-14 and the relative configurations of all the chiral centers in **2** was established by analysis of NOESY correlations as shown in Figure 3. Assuming that the β-orientation of H_3_-13 (δ 1.05, s) and H_3_-13 showed NOESY correlations with H_3_-15 (δ 0.87, d, *J* = 6.8 Hz), H-7β (δ 2.48, m), and H-2 (δ 5.29 brs), H_3_-15 and H-2 should also be positioned on the β-face. NOESY correlations observed from H-12 (δ 1.71 m) to H-4, H-6α (δ 1.83, m) to H-5 (δ 5.18, ddd, *J* = 9.2, 4.8, 3.2 Hz), and H-5 to H-4 (δ 6.63, d, *J* = 3.2 Hz) reflected the α-orientation of H-12, H-4, and H-5. Therefore, **2** was found to be the C-5 epimer of paralemnolin E [9].

Parathyrsoidin F (**3**) was obtained as a colorless oil. The HRESIMS of **3** established the molecular formula C_16_H_26_O_5_, implying four degrees of unsaturation. The DEPT spectrum (Table 2) of **3** evidenced four methyl, four sp^3^ methylene, three sp^3^ methine, two sp^3^ quaternary, and two carbonyl carbons. In turn, the ^1^H and ^13^C NMR (Table 2 and Table 3) (Figures S13 and S14) spectra showed the presence of (a) a secondary methyl (δ_C_ 15.5 CH_3_; δ_H_ 0.35 d, *J* = 7.2 Hz), (b) a tertiary methyl (δ_C_ 16.4 CH_3_; δ_H_ 0.54 s), (c) a secondary hydroxyl on C-1 (δ_C_ 71.7 CH; δ_H_ 3.32 brs), (d) a tertiary hydroxyl on C-10 (δ_C_ 72.6 C, (e) a COCH_3_ group at C-6 (δ_C_ 214.3 CO, 34.3 CH_3_; δ_H_ 1.82 s), and (f) an acetate at C-7 (δ_C_ 169.1 CO, 20.6 CH_3_; δ_H_ 1.52 s). IR absorption at 3421 cm^−^^1^ and two absorptions at 1736 and 1714 cm^−^^1^ and the NMR signals indicated the presence of a hydroxyl, an acetate, and a methyl ketone (Table 2 and Table 3). The structure of **3** was determined by COSY (Figure 2 and Figure S15) and HMBC correlations (Figure 2 and Figure S17), and the latter correlations determined the acetate to be at C-7 (71.6 CH). The relative configuration of **3** was established from NOESY cross-peaks (Figure 3 and Figure S18). Assuming the β-orientation of H_3_-14 (δ 0.54 s), which exhibited NOESY correlations with H-1 (δ 3.32, d, *J* = 5.2 Hz), H-6 (δ 2.99, d, *J* = 6.8 Hz), H-7 (δ 5.16, ddd, *J* = 12.4, 6.8, 4.8 Hz), and H_3_-13 (δ 0.35, d, *J* = 7.2 Hz), the β-orientation of H-1, H-6, H-7, and H_3_-14 were suggested (Figure 3). One of the methylene protons at C-2 (δ 1.75, m) exhibited a NOESY correlation with H-1 and was identified as H-2β, while the other (δ 1.92, m) was assigned to H-2α. The NOESY correlation observed between H-2α and 10-OH (δ 6.68, d, *J* = 1.2 Hz) indicated the α-orientation of the 10-OH.

The spectral evidence of parathyrsoidin G (**4**) (C_16_H_24_O_4_) suggested that this compound was a dehydrated form of **3**. ^1^H-^1^H COSY correlations (Figure S21) from H-6 to H-9 through H-7 and H_2_-8 and from H-1 to H_3_-14 through H_2_-2, H_2_-3, and H-4, together with HMBC correlations from H_3_-13 to C-4, C-5, C-6, C-10, H-6 to C-5, C-7, C-8, C-10, and H-9 to C-1 (Figure 2 and Figure S23) and NOESY correlations (Figure 3 and Figure S24) between H_3_-13/H-1, H_3_-13/H-6, H_3_-13/H-7, H-6/H-7, and H-6/H_3_-14, confirmed that **4** was a dehydrated analogue of **3**.

Cytotoxicity of Compounds **1**–**4** against the proliferation of a limited panel of cancer cell lines was evaluated against mouse lymphocytic leukemia (P-388), human colon adenocarcinoma (HT-29), and human lung epithelial carcinoma (A-549). Compounds **1**–**4** displayed cytotoxicity against P-388, with ED_50_ values of 3.18, 2.59, 3.31, and 2.49 μg/mL, respectively. However, Compounds **1**–**4** were not cytotoxic to A549 and HT-29 cell lines. Compounds **1**–**4** were also examined for the antiviral activity against human cytomegalovirus (HCMV), but none of them showed such activity.

## 3. Experimental Section

### 3.1. General Experimental Procedures

Optical rotations were determined with a JASCO P1020 digital polarimeter (Tokyo, Japan). UV and IR spectra were obtained on JASCO V-650 (Tokyo, Japan) and JASCO FT/IR-4100 spectrophotometers (Tokyo, Japan), respectively. NMR spectra were recorded on a Varian MR 400 NMR spectrometer (Santa Clara, CA, USA) at 400 MHz for ^1^H and 100 MHz for ^13^C. ^1^H NMR chemical shifts are expressed in δ (ppm) referring to the solvent peak δ_H_ 7.27 for CHCl_3_ or δ_H_ 7.15 for C_6_D_6_, and coupling constants are expressed in Hertz (Hz). ^13^C NMR chemical shifts are expressed in δ (ppm) referring to the solvent peak δ_C_ 77.0 for CDCl_3_ or δ_C_ 128.0 for C_6_D_6_. MS were recorded by a Bruker APEX II mass spectrometer (Bruker, Bremen, Germany). Silica gel 60 (Merck, Darmstadt, Germany, 230–400 mesh) and LiChroprep RP-18 (Merck, 40–63 µm) were used for column chromatography. Precoated silica gel plates (Merck, Kieselgel 60 F_254_, 0.25 mm) and precoated RP-18 F_254s_ plates (Merck) were used for thin-layer chromatography (TLC) analysis. High-performance liquid chromatography (HPLC) (Tokyo, Japan) was carried out using a Hitachi L-7100 pump (Tokyo, Japan) equipped with a Hitachi L-7400 UV detector (Tokyo, Japan) at 220 nm together with a semi-preparative reversed-phased column (Merck, Hibar LiChrospher RP-18e, 5 µm, 250 mm × 25 mm).

### 3.2. Animal Material

The octocoral *N. chabroli* was collected by hand using scuba at Green Islang, Taitong County, Taiwan, in August 2015, at a depth of 6 m. A voucher specimen (GN-100) was deposited in the Department of Marine Biotechnology and Resources, National Sun Yat-sen University.

The octocoral *P. thyrsoides* was collected by hand using scuba at San-Hsian-Tai, Taitong County, Taiwan, in July 2008, at a depth of 6 m. A voucher specimen (SST-07) was deposited in the Department of Marine Biotechnology and Resources, National Sun Yat-sen University.

### 3.3. Extraction and Separation

The frozen soft coral was chopped into small pieces and extracted with acetone in a percolator at room temperature. The acetone extract of *N. chabroli* was concentrated to a brown gum, which was partitioned with EtOAc and H_2_O. The EtOAc-soluble residue (50 g) was subjected to Si 60 CC using *n*-hexane–EtOAc mixtures of increasing polarity for elution. Fraction 10, eluted with *n*-hexane–EtOAc (1:10), was purified by reverse-phase HPLC (MeOH–H_2_O, 85:15) to afford **1** (2.9 mg).

The frozen soft coral was chopped into small pieces and extracted with acetone in a percolator at room temperature. The acetone extract of *P. thyrsoides* was concentrated to a brown gum, which was partitioned with EtOAc and H_2_O. The EtOAc-soluble residue (10 g) was subjected to Si 60 CC using *n*-hexane–EtOAc mixtures of increasing polarity for elution. Fraction 8, eluted with *n*-hexane–EtOAc (30:1), was purified by reverse-phase HPLC (MeOH–H_2_O, 95:5) to afford **2** (3.0 mg). Fraction 15, eluted with *n*-hexane–EtOAc (1:1), was purified by reverse-phase HPLC (MeOH–H_2_O, 55:45) to afford **3** (3.0 mg) and **4** (1.0 mg).

*Chabrolin A* (**1**): Colorless amorphous solid; ${\left[\alpha \right]}_{D}^{25}$ = +102 (*c* 0.1, CHCl_3_); IR (neat) ν_max_ 3439, 2965, 1635, and 772 cm^−1^; ^1^H NMR data, see Table 1; ^13^C NMR data, see Table 2; ESIMS *m*/*z* 311 [M + Na]^+^; (+)-HRESIMS *m*/*z* 311.23465 (calcd. for C_20_H_32_ONa, 311.23454).

*Parathyrsoidin E* (**2**): Colorless amorphous solid; ${\left[\alpha \right]}_{D}^{25}$ = −25 (*c* 0.3, CHCl_3_); IR (neat) ν_max_ 2964, 2927, 1743, 1455, 1369, 1247, and 871 cm^−1^; ^1^H NMR data, see Table 1; ^13^C NMR data, see Table 2; ESIMS *m*/*z* 343 [M + Na]^+^; (+)-HRESIMS *m*/*z* 343.1887 (calcd. for C_19_H_28_O_4_Na, 343.1885).

*Parathyrsoidin F* (**3**): Colorless amorphous solid; ${\left[\alpha \right]}_{D}^{25}$ = −54 (*c* 0.3, CHCl_3_); IR (neat) ν_max_ 3421, 2926, 1736, 1714, 1376, 1245, 1111, and 859 cm^−1^; ^1^H NMR data, see Table 1; ^13^C NMR data, see Table 2; ESIMS *m*/*z* 321 [M + Na]^+^; (+)-HRESIMS *m*/*z* 321.1676 (calcd. for C_16_H_26_O_5_Na, 321.1678).

*Parathyrsoidin G* (**4**): Colorless amorphous solid; ${\left[\alpha \right]}_{D}^{25}$ = −87 (*c* 0.1, CHCl_3_); IR (neat) ν_max_ 3421, 2964, 1739, 1714, 1362, 1236, and 869 cm^−1^; ^1^H NMR data, see Table 1; ^13^C NMR data, see Table 2; ESIMS *m*/*z* 303 [M + Na]^+^; (+)-HRESIMS *m*/*z* 303.1573 (calcd for C_16_H_24_O_4_Na, 303.1572).

### 3.4. Cytotoxicity Assay

Cytotoxicity was determined on P-388 (mouse lymphocytic leukemia), HT-29 (human colon adenocarcinoma), and A-549 (human lung epithelial carcinoma) tumor cells using a modification of the MTT colorimetric method according to a previously described procedure [20,21]. The provision of the P-388 cell line was supported by J.M. Pezzuto, formerly of the Department of Medicinal Chemistry and Pharmacognosy, University of Illinois at Chicago. HT-29 and A-549 cell lines were purchased from the American Type Culture Collection. To measure the cytotoxic activities of tested compounds, five concentrations with three replications were performed on each cell line. Mithramycin was used as a positive control.

### 3.5. Anti-HCMV Assay

To determine the effects of natural products upon the HCMV cytopathic effect (CPE), confluent human embryonic lung (HEL) cells grown in 24-well plates were incubated for 1 h in the presence or absence of various concentrations of tested natural products with three replications. Ganciclovir was used as a positive control. Then, cells were infected with HCMV at an input of 1000 pfu (plaque forming units) per well of a 24-well dish. Antiviral activity was expressed as IC_50_ (50% inhibitory concentration), or a compound concentration required to reduce virus-induced CPE by 50% after 7 days as compared with the untreated control. To monitor the cell growth upon treating with natural products, an MTT-colorimetric assay was employed [22].

## 4. Conclusions

**Scheme 1:**
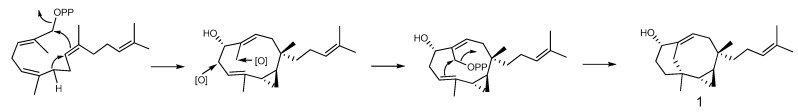
A plausible biosynthetic pathway of **1**.

Cytotoxicity-guided fractionation of the ethyl acetate extract of the soft corals *N. chablroli* and *P. thyrsoides* led to the isolation of a novel diterpenoid, chabrolin A (**1**) (possessing an unprecedented terpenoid skeleton) as well as three new sesquiterpenoids. Compounds **1**–**4** displayed cytotoxicity against P-388, with ED_50_ values of 3.18, 2.59, 3.31, and 2.49 μg/mL, respectively. However, Compounds **1**–**4** were not cytotoxic to A549 and HT-29 cell lines and did not show anti-HCMV activity. A plausible biosynthetic pathway of **1** is proposed in Scheme 1.

## Figures and Tables

**Table 1:** NMR spectral data of **1**.

Position	δ_H_ *^a^* (*J* in Hz)	δ_C_ *^b^*, Type	COSY	HMBC	NOESY
1	1.62 s	17.6, CH_3_	3	2, 3, 17	-
2	-	130.9, C	-	-	-
3	5.13 t (7.2)	125.3, CH	1, 4, 17	1, 17	17
4	1.86 m; 2.04 m	22.8, CH_2_	3, 5	-	-
5	1.35 m	45.3, CH_2_	4	3, 6, 7, 16, 18	16
6	-	39.7, C	-	-	-
7a	2.24 m	40.9, CH_2_	8	6, 8, 9, 16	16
7b	1.79 m		8	6, 8, 9, 10	8, 18
8	5.59 t (8.0)	121.6, CH	7	6, 7, 10	10, 18
9	-	144.5, C	-	-	-
10	4.29 s	74.1, CH	11, 19	8	8, 11b
11a	2.05m	32.4, CH_2_	12	10,	-
11b	1.86 m		12	10,	14
12	1.41 m; 1.77 m	33.6, CH_2_	11	-	-
13	-	37.4, C	-	-	-
14	0.78 dd (14.8, 7.2)	15.6, CH	15, 16	12, 13, 16, 20	11b, 15b, 18
15a	0.49 m	8.6, CH_2_	14, 16	6, 13, 16	20
15b	0.36 m		14, 16	6, 13, 16	14, 18,
16	0.25 dd (15.2, 7.2)	23.7, CH	14, 15	-	5, 20
17	1.69 s	25.7, CH_3_	3	1, 2, 3	3
18	0.52 s	15.9, CH_3_	-	5, 6, 7, 16	8, 10, 14, 15a
19a	2.08 m	38.9, CH_2_	10	8, 9, 10, 13, 14	16, 20
19b	2.16 m		10	8, 9, 10, 13, 14	20
20	0.64 s	23.2, CH_3_	-	12, 13, 14, 19	15a, 16, 19

*^a^* Spectra were measured in CDCl_3_ (400 MHz). *^b^* Spectra were measured in CDCl_3_ (100 MHz).

**Table 2:** ^13^C NMR spectral data of **2**–**4**.

Position	2, δ_H_ *^a^* (Type)	3, δ_H_ *^b^* (Type)	4, δ_H_ *^b^* (Type)
1	43.2, C	71.7, CH	68.9, CH
2	137.9, CH	30.86, CH_2_	35.8, CH_2_
3	128.0, C	29.0, CH_2_	28.7, CH_2_
4	71.0, CH	30.94, CH	35.4, CH
5	74.7, CH	46.1, C	43.9, C
6	30.2, CH_2_	59.5, CH	58.5, CH
7	31.2, CH_2_	71.6, CH	69.4, CH
8	141.0, C	23.1, CH_2_	27.4, CH_2_
9	125.9, CH	29.6, CH_2_	114.0, CH
10	23.1, CH_2_	72.6, C	143.6, C
11	26.2, CH_2_	214.3, C	206.9, C
12	38.3, CH	34.3, CH_3_	33.8, CH_3_
13	23.6, CH_3_	15.5, CH_3_	15.3, CH_3_
14	21.4, CH_3_	16.4, CH_3_	21.1, CH_3_
15	15.0, CH_3_	-	-
4-OAc	170.3, C 21.2, CH_3_	-	-
5-OAc	170.1, C 20.8, CH_3_	-	-
7-OAc	-	169.1, C 20.6, CH_3_	169.0, C 20.4, CH_3_

*^a^* Spectra were measured in CDCl_3_ (100 MHz). *^b^* Spectra were measured in C_6_D_6_ (100 MHz).

**Table 3:** ^1^H NMR spectral data of **2**–**4**.

Position	2, δ_H_ *^a^* (*J* in Hz)	3, δ_H_ *^b^* (*J* in Hz)	4, δ_H_ *^b^* (*J* in Hz)
1	-	3.52 d (5.2)	3.76 m
2	5.29 brs	α: 1.92 m; β: 1.75 m	α: 1.13 m; β: 1.73 m
3	-	α: 1.16 m; β: 0.98 m	α: 1.13 m; β: 1.04 m
4	6.63 d (3.2)	1.86 m,	1.63 m
5	5.18 ddd (9.2, 4.8, 3.2)	-	-
6	α: 1.83 m; β: 1.94 m	2.99 d (6.8)	3.09 d (5.2)
7	α: 2.10 ddd (14.8, 12.4, 4.4); β: 2.48 m	5.16 ddd (12.4, 6.8, 4.8)	5.38 ddd (10.0, 6.4, 5.2)
8	-	α: 1.92 m; β: 1.64 ddt (12.4, 4.0, 4.0)	α: 2.42 m; β: 2.34 m
9	5.57 dd (3.6, 3.2)	α: 2.22 ddd (14.0, 4.0, 4.0); β: 1.42 td (14.0, 4.0)	5.67 dd (6.4, 2.8)
10	2.03 m	-	-
11	α: 1.78 m; β: 1.39 m	-	-
12	1.71 m	1.82 s	1.96 s
13	1.05 s	0.35 d (7.2)	0.54 d (6.8)
14	1.68 d (1.2)	0.54 s	0.82 s
15	0.87 d (6.8)	-	-
4-OAc	2.06 s	-	-
5-OAc	2.03 s	-	-
7-OAc	-	1.52 s	1.63 s
12-OH	-	6.68 d (1.2)	-

*^a^* Spectra were measured in CDCl_3_ (400 MHz). *^b^* Spectra were measured in C_6_D_6_ (400 MHz).

## Supplementary Materials

NMR spectra of new compounds **1**–**4** are available online at www.mdpi.com/1660-3397/15/12/392/s1, Figure S1. 1H NMR spectrum (400 MHz) of chabrolin A (1) in CDCl3; Figure S2. 13C NMR spectrum (100 MHz) of chabrolin A (1) in CDCl3; Figure S3. COSY spectrum (400 MHz) of chabrolin A (1) in CDCl3; Figure S4. HSQC spectrum (400 MHz) of chabrolin A (1) in CDCl3; Figure S5. HMBC spectrum (400 MHz) of chabrolin A (1) in CDCl3; Figure S6. NOESY spectrum (400 MHz) of chabrolin A (1) in CDCl3; Figure S7. 1H NMR spectrum (400 MHz) of parathyrsoidin E (2) in CDCl3; Figure S8. 13C NMR spectrum (100 MHz) of parathyrsoidin E (2) in CDCl3; Figure S9. COSY spectrum (400 MHz) of parathyrsoidin E (2) in CDCl3; Figure S10. HSQC spectrum (400 MHz) of parathyrsoidin E (2) in CDCl3; Figure S11. HMBC spectrum (400 MHz) of parathyrsoidin E (2) in CDCl3; Figure S12. NOESY spectrum (400 MHz) of parathyrsoidin E (2) in CDCl3; Figure S13. 1H NMR spectrum (400 MHz) of parathyrsoidin F (3) in C6D6; Figure S14. 13C NMR spectrum (100 MHz) of parathyrsoidin F (3) in C6D6; Figure S15. COSY spectrum (400 MHz) of parathyrsoidin F (3) in C6D6; Figure S16. HSQC spectrum (400 MHz) of parathyrsoidin F (3) in C6D6; Figure S17. HMBC spectrum (400 MHz) of parathyrsoidin F (3) in C6D6; Figure S18. NOESY spectrum (400 MHz) of parathyrsoidin F (3) in C6D6; Figure S19. 1H NMR spectrum (400 MHz) of parathyrsoidin G (4) in C6D6; Figure S20. 13C NMR spectrum (100 MHz) of parathyrsoidin G (4) in C6D6; Figure S21. COSY spectrum (400 MHz) of parathyrsoidin G (4) in C6D6; Figure S22. HSQC spectrum (400 MHz) of parathyrsoidin G (4) in C6D6; Figure S23. HMBC spectrum (400 MHz) of parathyrsoidin G (4) in C6D6; Figure S24. NOESY spectrum (400 MHz) of parathyrsoidin G (4) in C6D6.
