# Supplementary material for: New Cytotoxic Terpenoids from Soft Corals Nephthea chabroli and Paralemnalia thyrsoides
Source: Mar Drugs. 2017 Dec 19;15(12):392. doi: 10.3390/md15120392 (PMC5742852; doi:10.3390/md15120392)

Supplementary Materials

Table of Contents

Figure S1. ^{1}H NMR spectrum (400 MHz) of chabrolin A (**1**) in $CDCl_3$. 2

Figure S2. ^{13}C NMR spectrum (100 MHz) of chabrolin A (**1**) in $CDCl_3$. 3

Figure S3. COSY spectrum (400 MHz) of chabrolin A (**1**) in $CDCl_3$. 4

Figure S4. HSQC spectrum (400 MHz) of chabrolin A (**1**) in $CDCl_3$. 5

Figure S5. HMBC spectrum (400 MHz) of chabrolin A (**1**) in $CDCl_3$. 6

Figure S6. NOESY spectrum (400 MHz) of chabrolin A (**1**) in $CDCl_3$ 7

Figure S7. ^{1}H NMR spectrum (400 MHz) of parathyrsoidin E (**2**) in $CDCl_3$. 8

Figure S8. ^{13}C NMR spectrum (100 MHz) of parathyrsoidin E (**2**) in $CDCl_3$ 9

Figure S9. COSY spectrum (400 MHz) of parathyrsoidin E (**2**) in $CDCl_3$ 10

Figure S10. HSQC spectrum (400 MHz) of parathyrsoidin E (**2**) in $CDCl_3$. 11

Figure S11. HMBC spectrum (400 MHz) of parathyrsoidin E (**2**) in $CDCl_3$. 12

Figure S12. NOESY spectrum (400 MHz) of parathyrsoidin E (**2**) in $CDCl_3$ 13

Figure S13. ^{1}H NMR spectrum (400 MHz) of parathyrsoidin F (**3**) in C_6D_6. 14

Figure S14. ^{13}C NMR spectrum (100 MHz) of parathyrsoidin F (**3**) in C_6D_6. 15

Figure S15. COSY spectrum (400 MHz) of parathyrsoidin F (**3**) in C_6D_6. 16

Figure S16. HSQC spectrum (400 MHz) of parathyrsoidin F (**3**) in C_6D_6. 17

Figure S17. HMBC spectrum (400 MHz) of parathyrsoidin F (**3)** in C_6D_6. 18

Figure S18. NOESY spectrum (400 MHz) of parathyrsoidin F (**3**) in C_6D_6. 19

Figure S19. ^{1}H NMR spectrum (400 MHz) of parathyrsoidin G (**4**) in C_6D_6. 20

Figure S20. ^{13}C NMR spectrum (100 MHz) of parathyrsoidin G (**4**) in C_6D_6. 21

Figure S21. COSY spectrum (400 MHz) of parathyrsoidin G (**4)** in C_6D_6. 22

Figure S22. HSQC spectrum (400 MHz) of parathyrsoidin G (**4**) in C_6D_6. 23

Figure S23. HMBC spectrum (400 MHz) of parathyrsoidin G (**4**) in C_6D_6. 24

Figure S24. NOESY spectrum (400 MHz) of parathyrsoidin G (**4**) in C_6D_6. 25

Figure S1. ^{1}H NMR spectrum (400 MHz) of chabrolin A (**1**) in $CDCl_3$

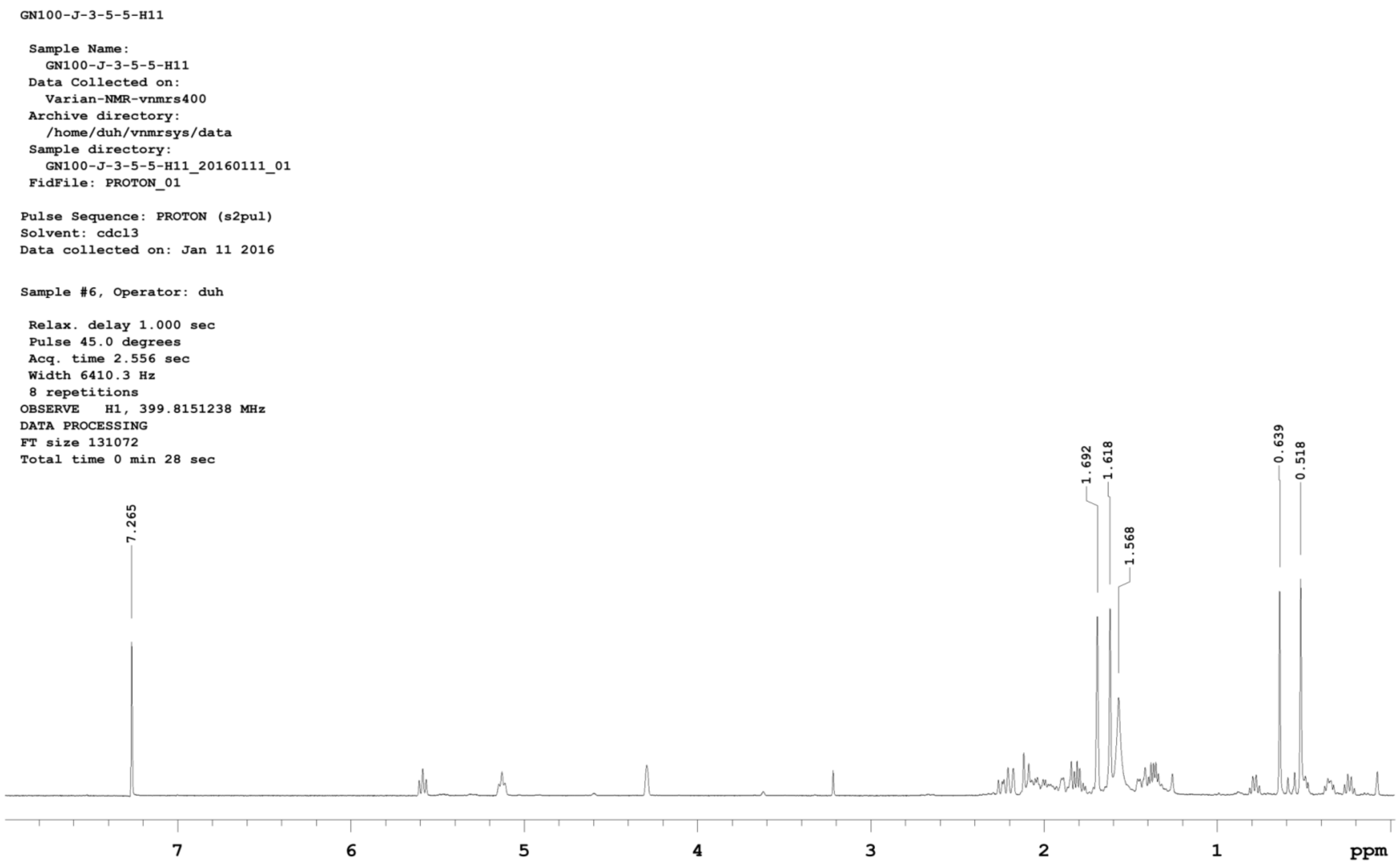

Figure S2. ^{13}C NMR spectrum (100 MHz) of chabrolin A (**1**) in $CDCl_3$

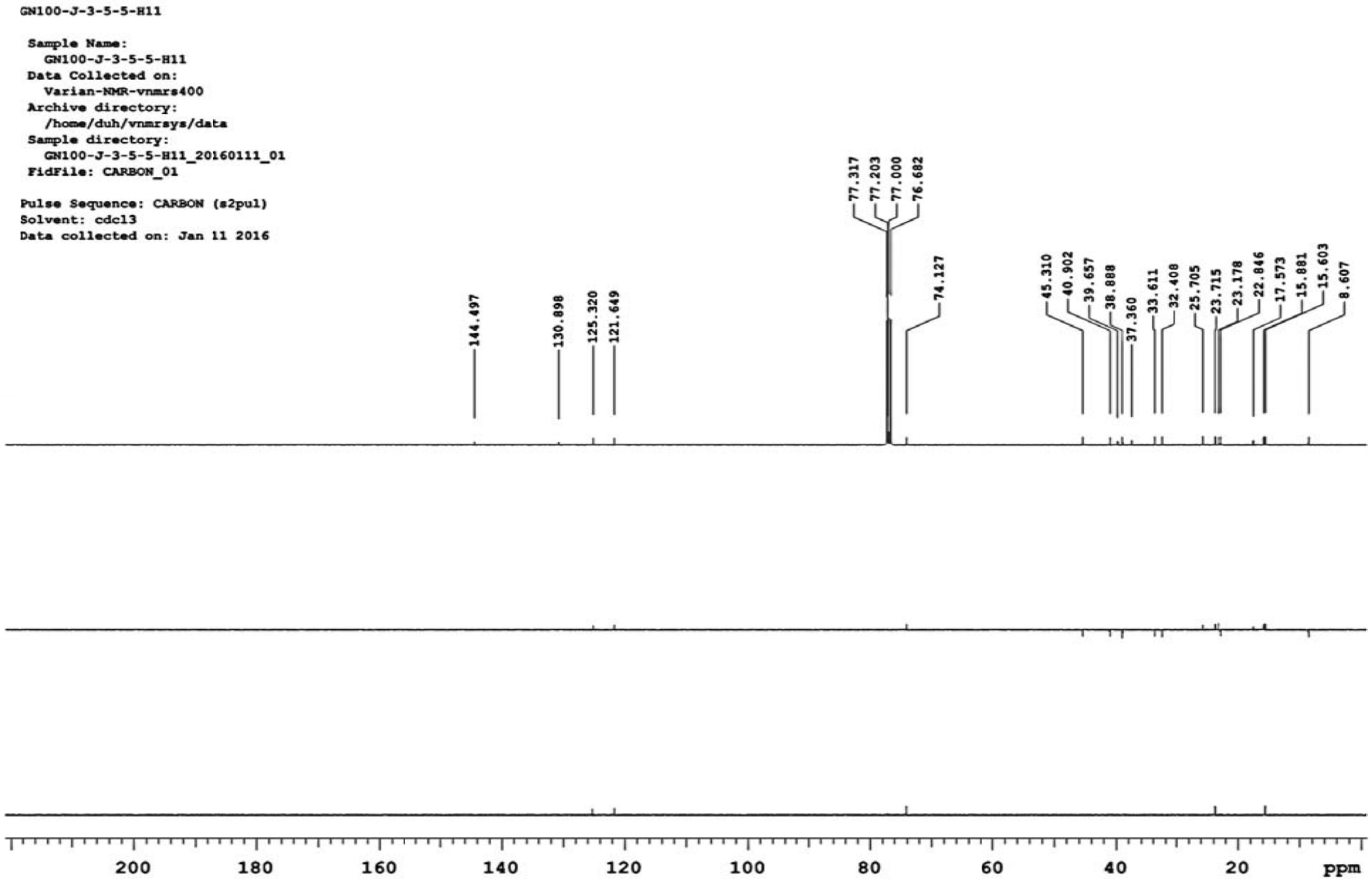

Figure S3. COSY spectrum (400 MHz) of chabrolin A (**1**) in $CDCl_3$

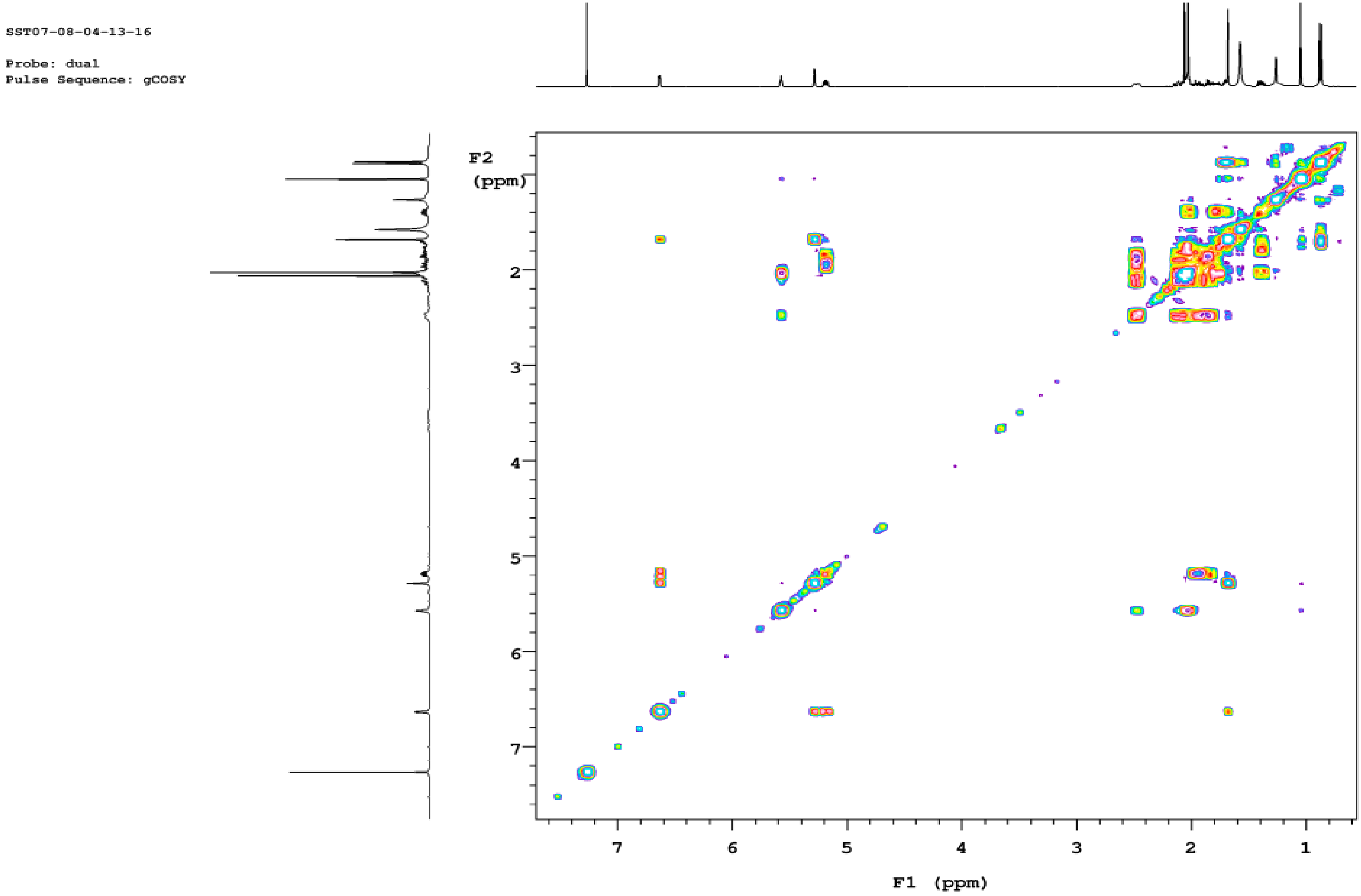

Figure S4. HSQC spectrum (400 MHz) of chabrolin A (**1**) in $CDCl_3$

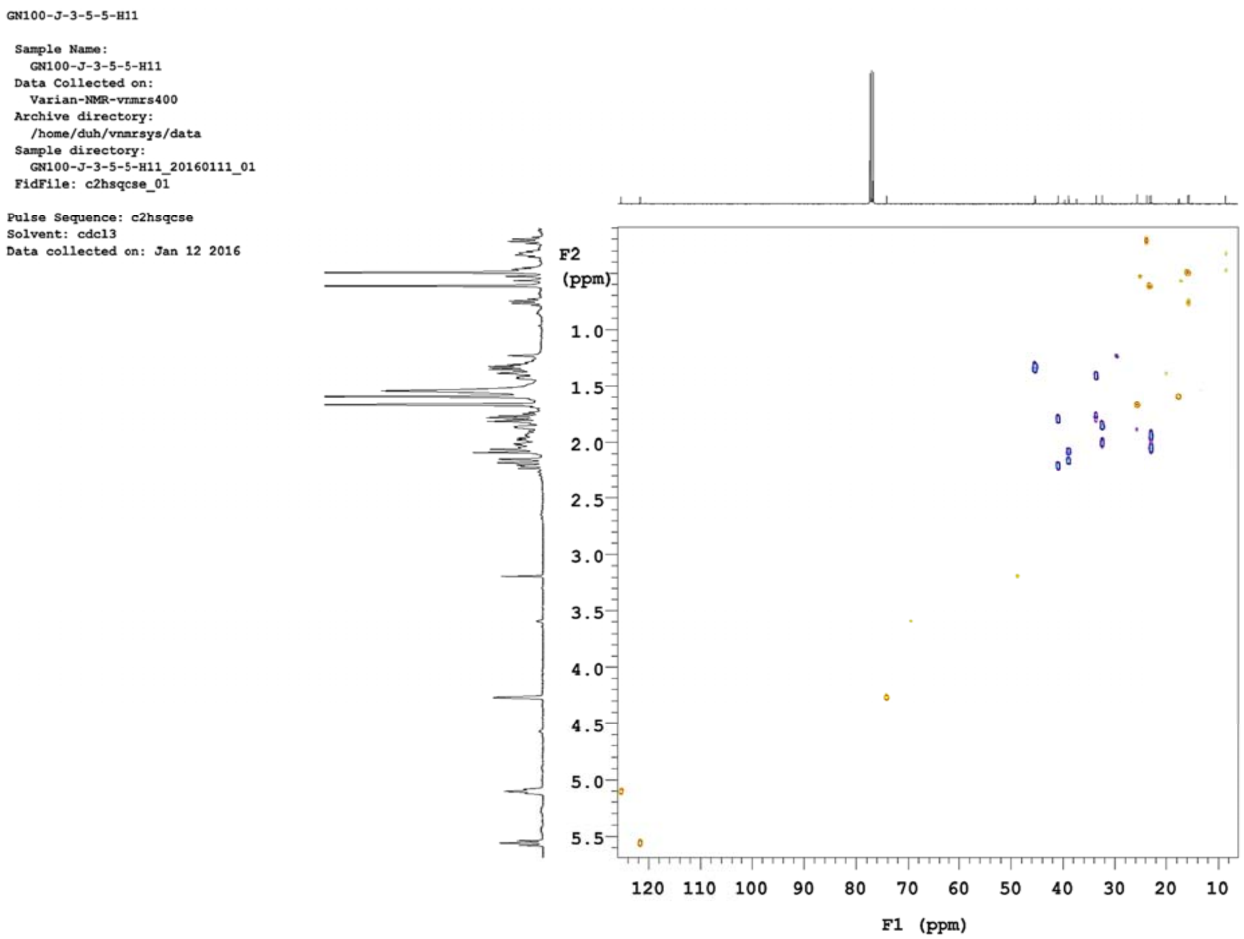

Figure S5. HMBC spectrum (400 MHz) of chabrolin A (**1**) in $CDCl_3$

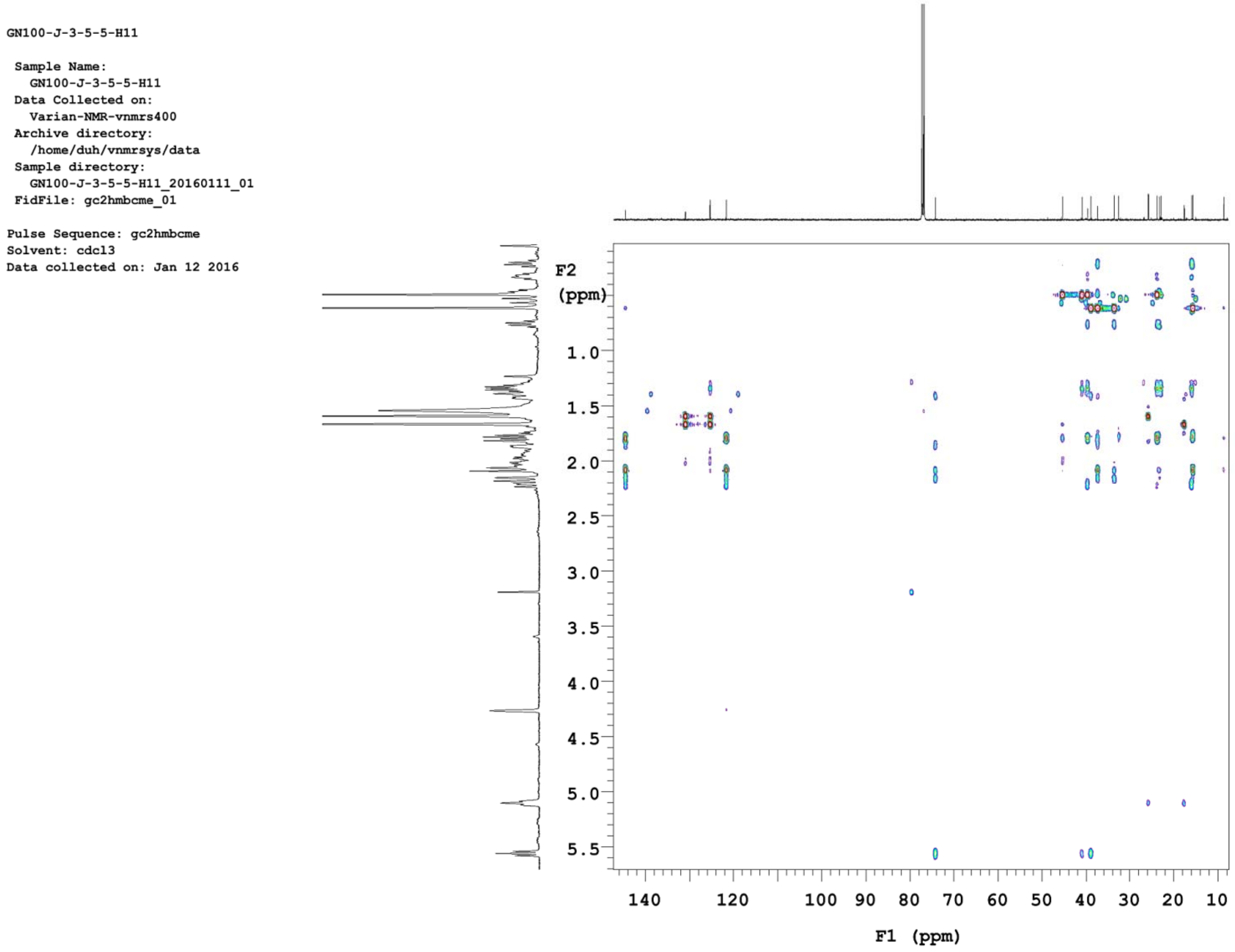

Figure S6. NOESY spectrum (400 MHz) of chabrolin A (**1**) in $CDCl_3$

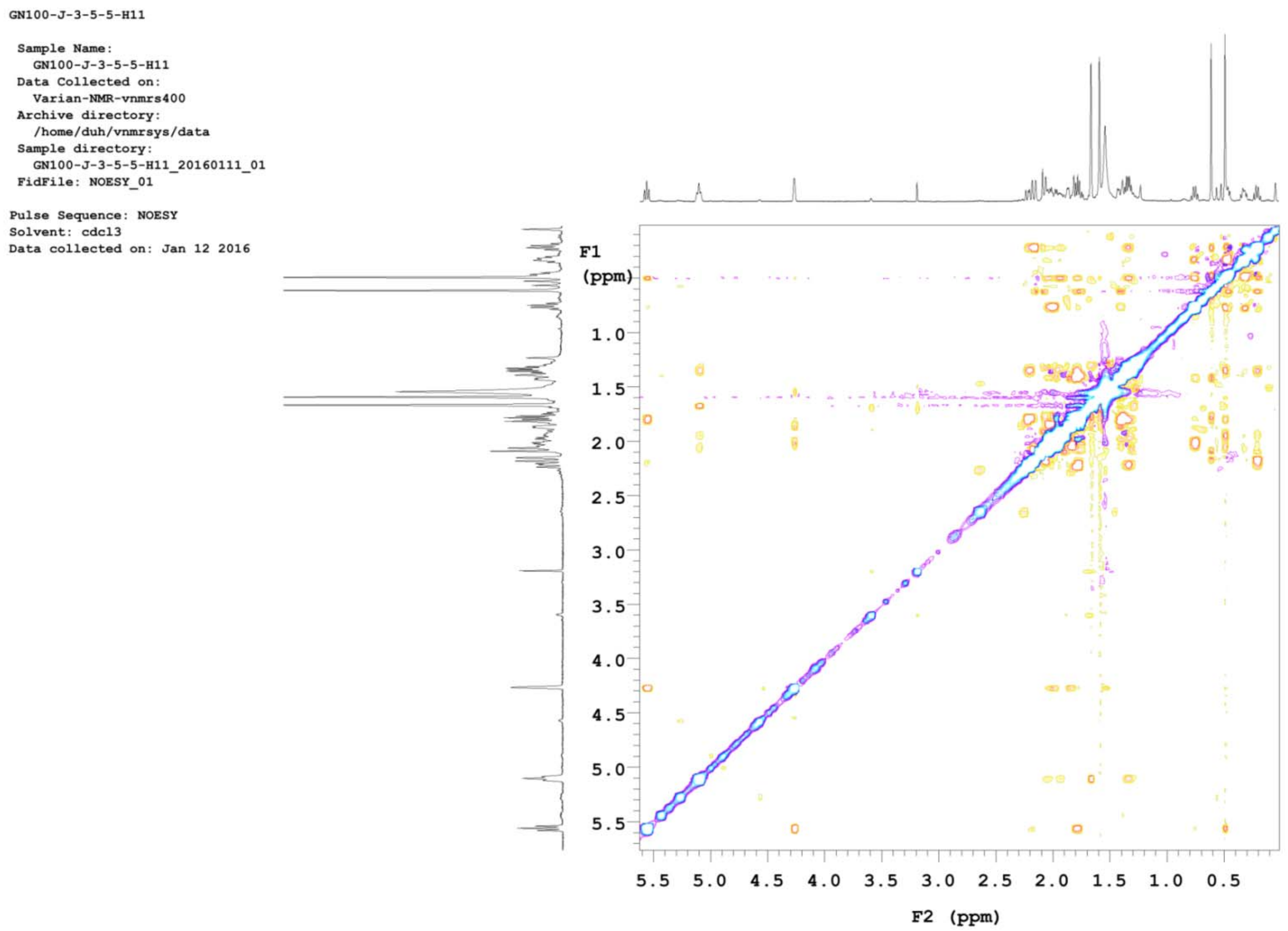

Figure S7. ^{1}H NMR spectrum (400 MHz) of parathyrsoidin E (**2**) in $CDCl_3$

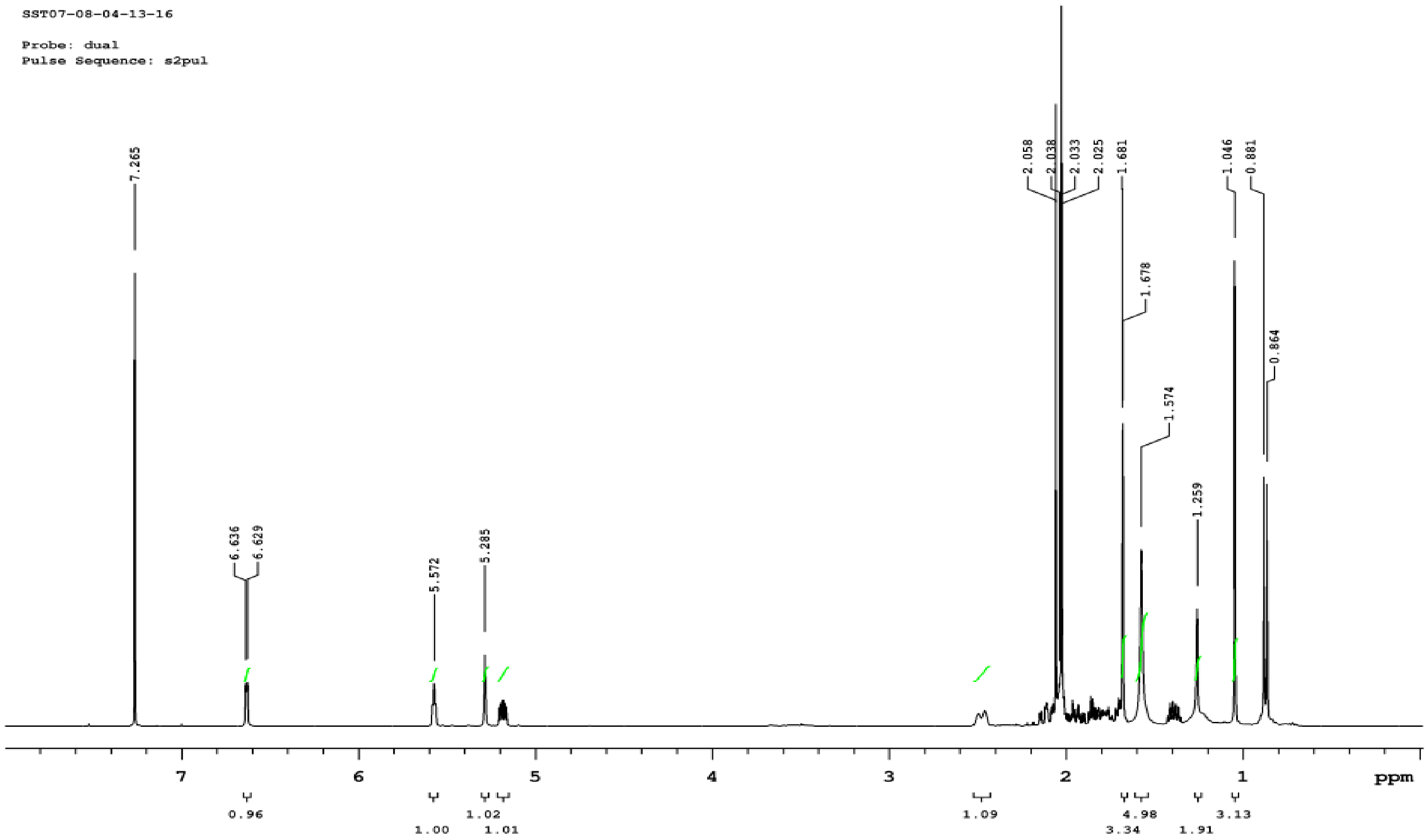

Figure S8. ^{13}C NMR spectrum (100 MHz) of parathyrsoidin E (**2**) in $CDCl_3$.

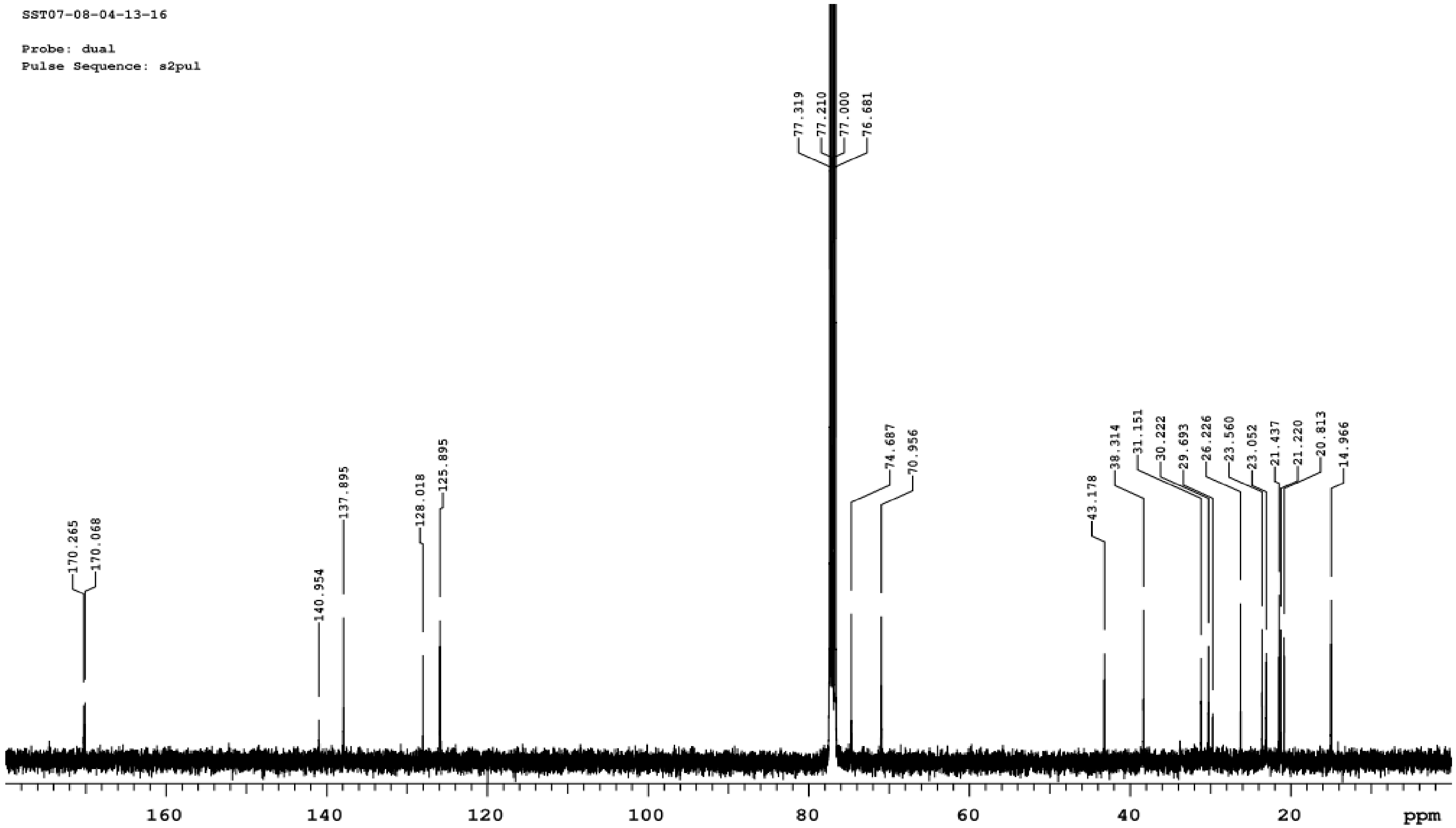

Figure S9. COSY spectrum (400 MHz) of parathyrsoidin E (**2**) in $CDCl_3$.

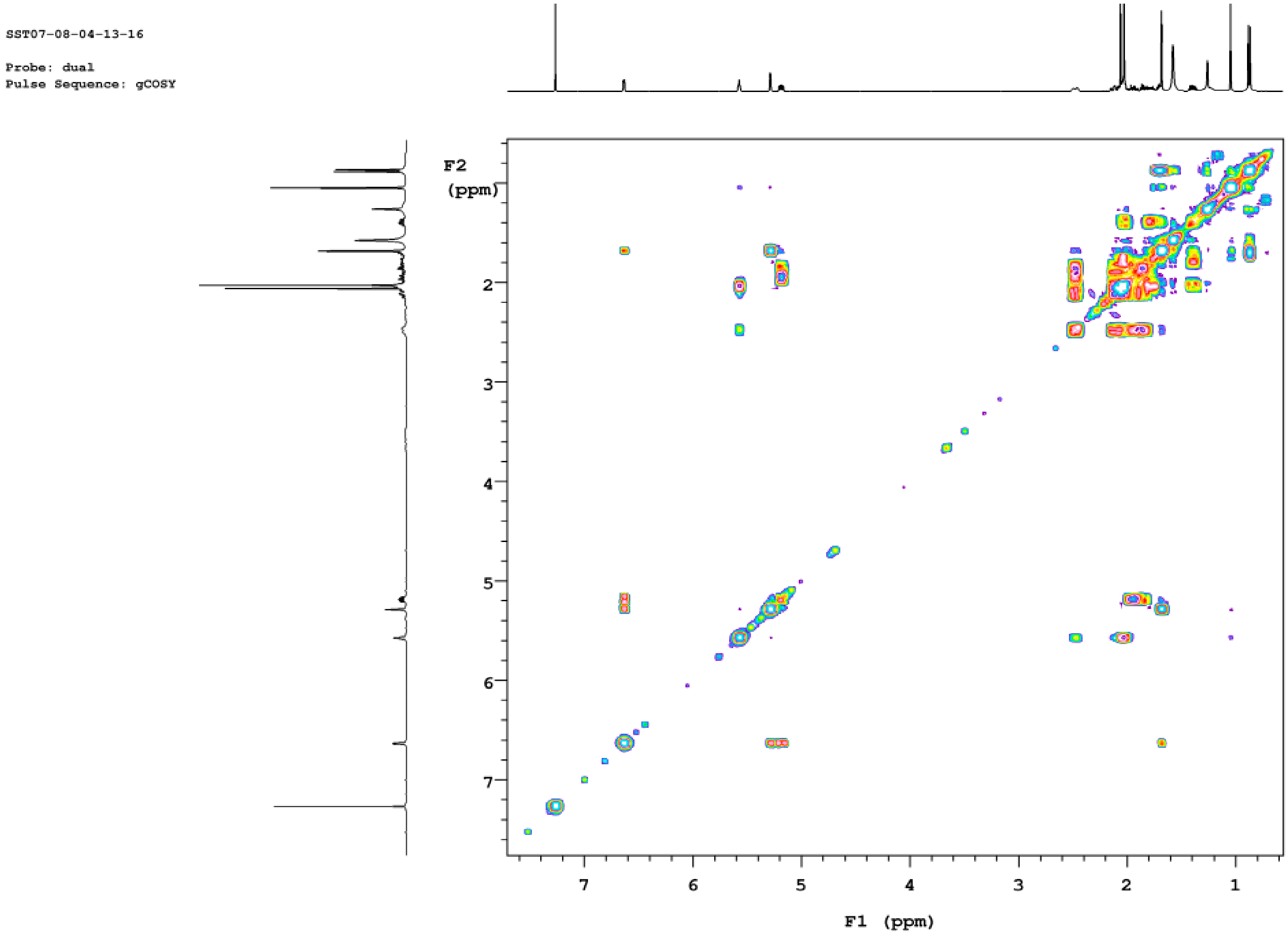

Figure S10. HSQC spectrum (400 MHz) of parathyrsoidin E (**2**) in $CDCl_3$.

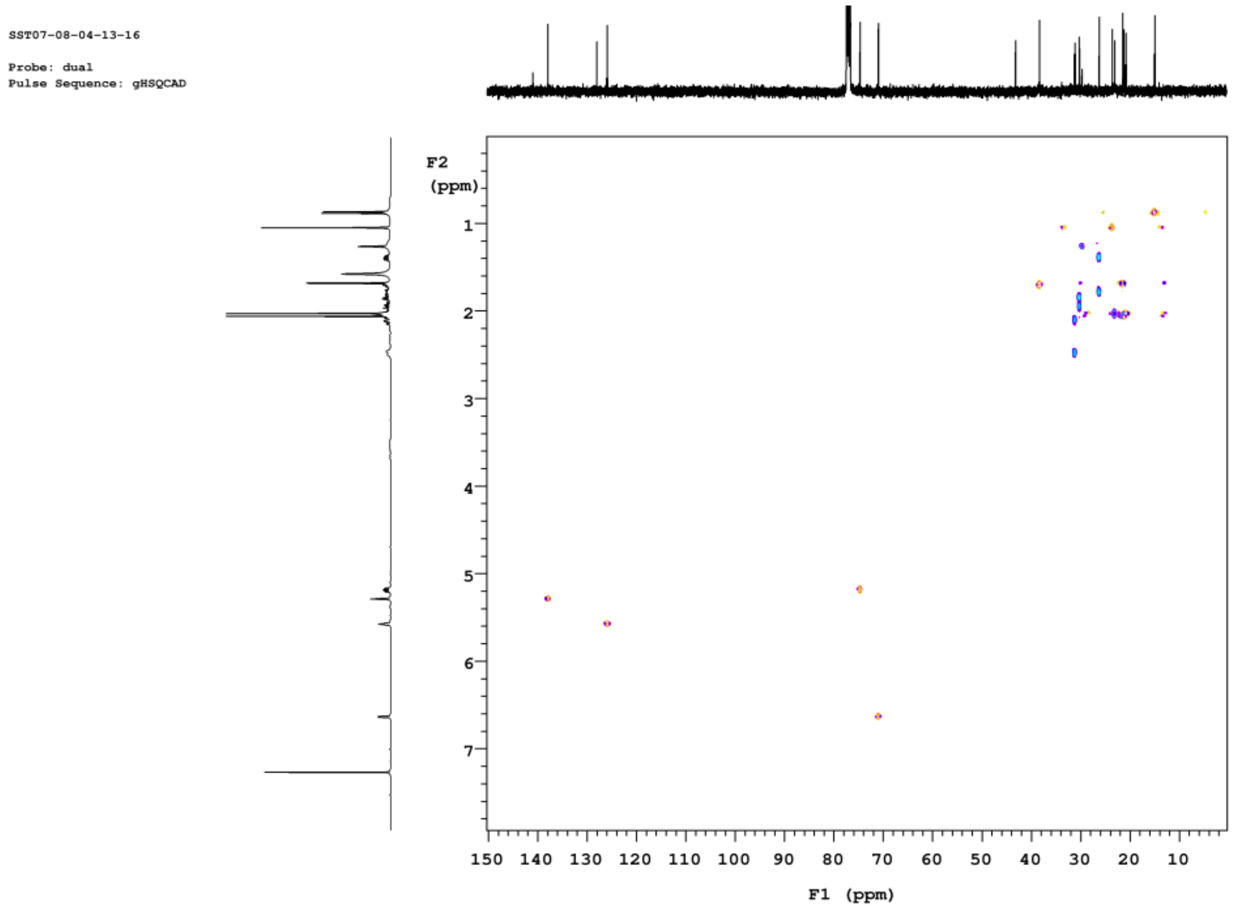

Figure S11. HMBC spectrum (400 MHz) of parathyrsoidin E (**2**) in $CDCl_3$.

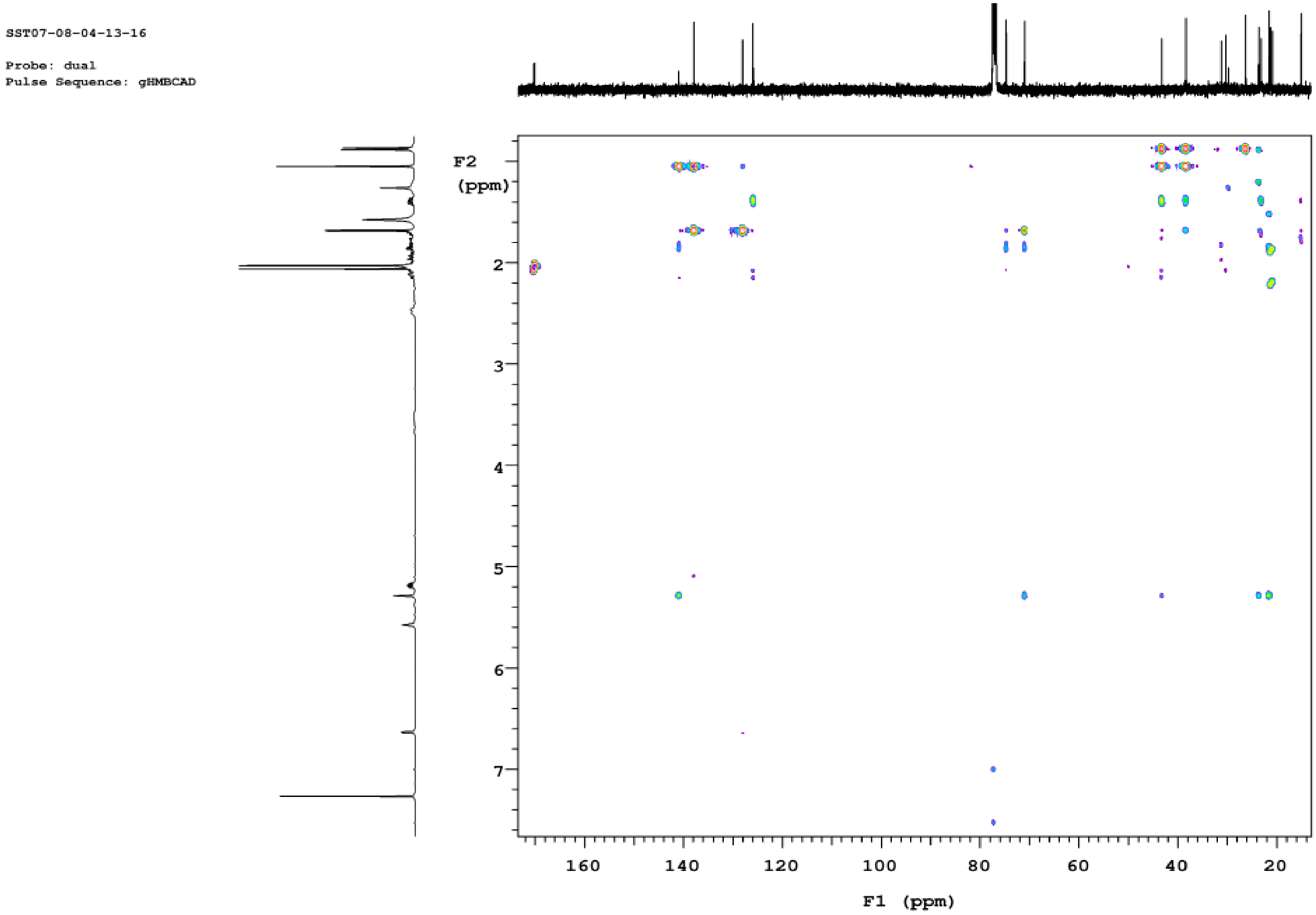

Figure S12. NOESY spectrum (400 MHz) of parathyrsoidin E **(2)** in $CDCl_3$.

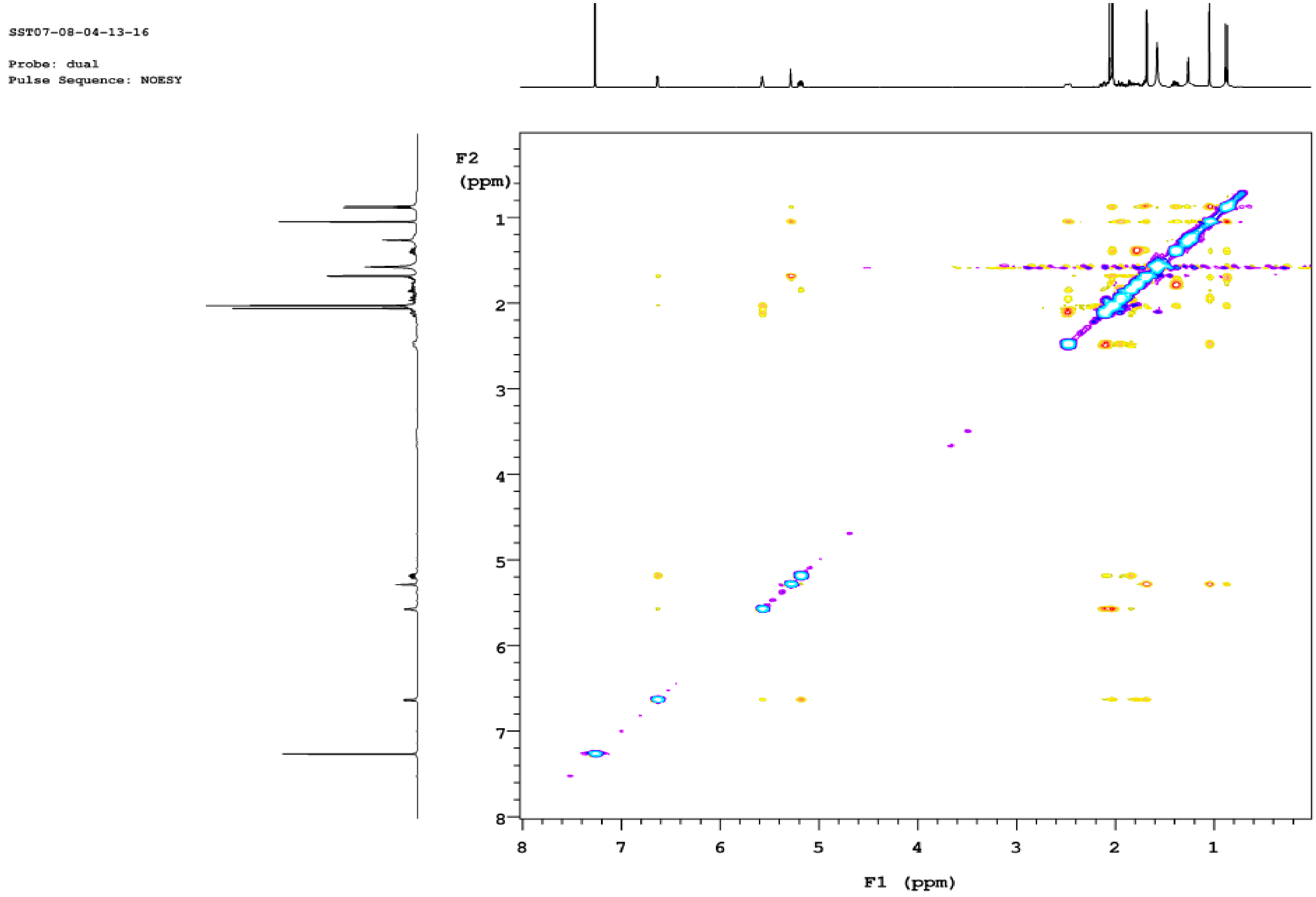

Figure S13^{1}H NMR spectrum (400 MHz) of parathyrsoidin F (**3**) n C_6D_6.

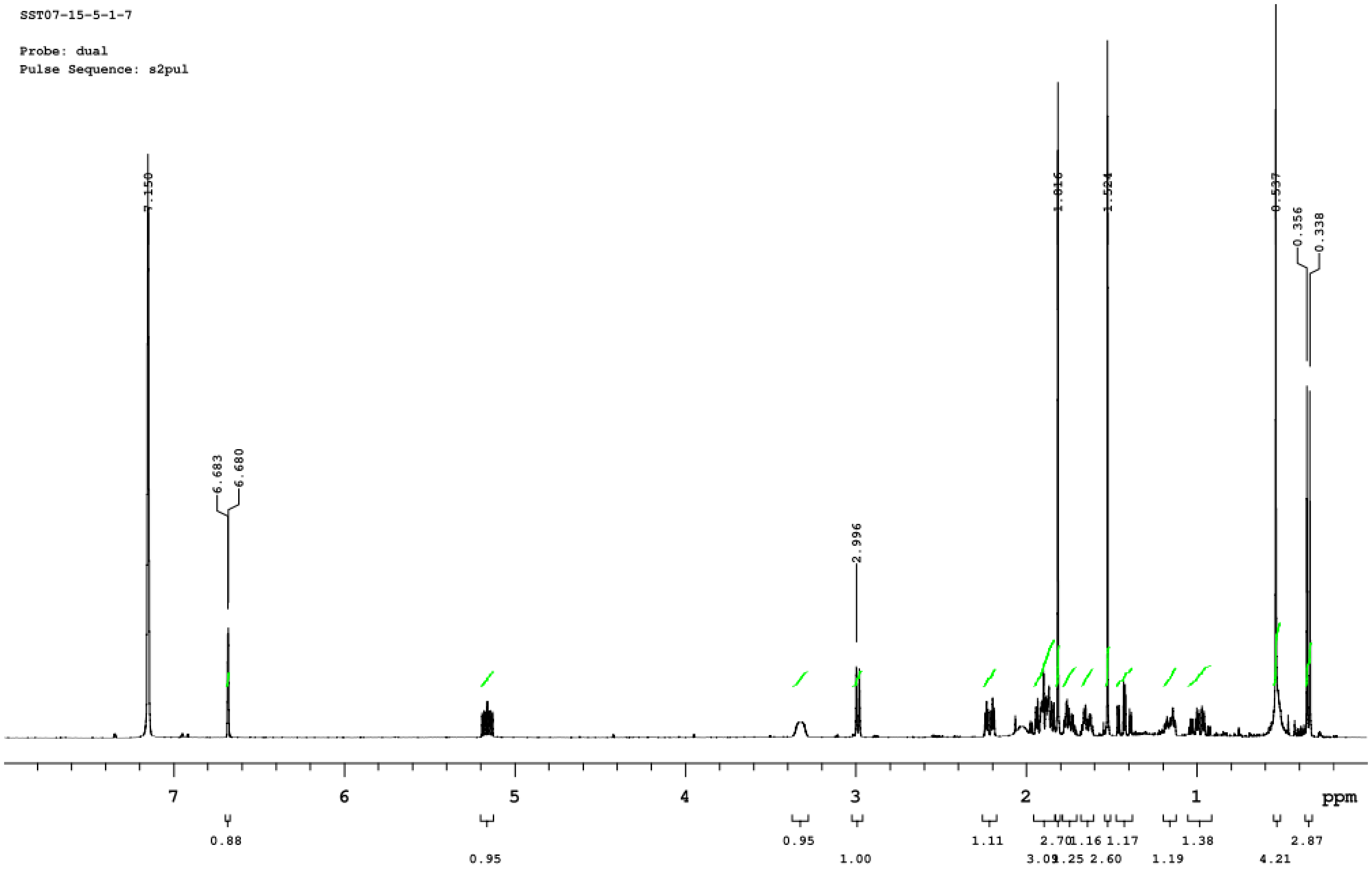

Figure S14 ^{13}C NMR spectrum (100 MHz) of parathyrsoidin F (**3**) in C_6D_6.

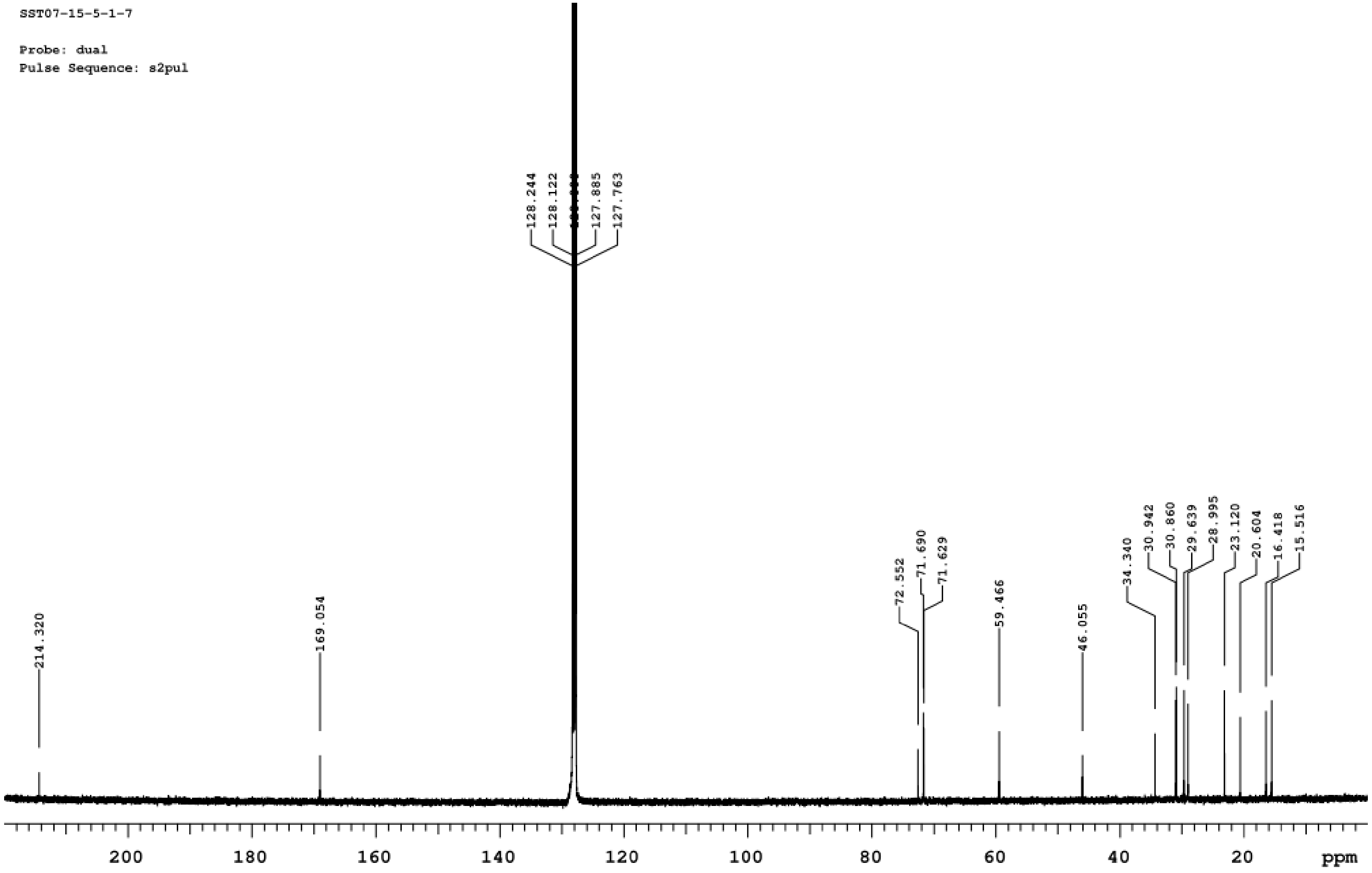

Figure S15. COSY spctrum (400 MHz) of parathyrsoidin F (**3**) in C_6D_6.

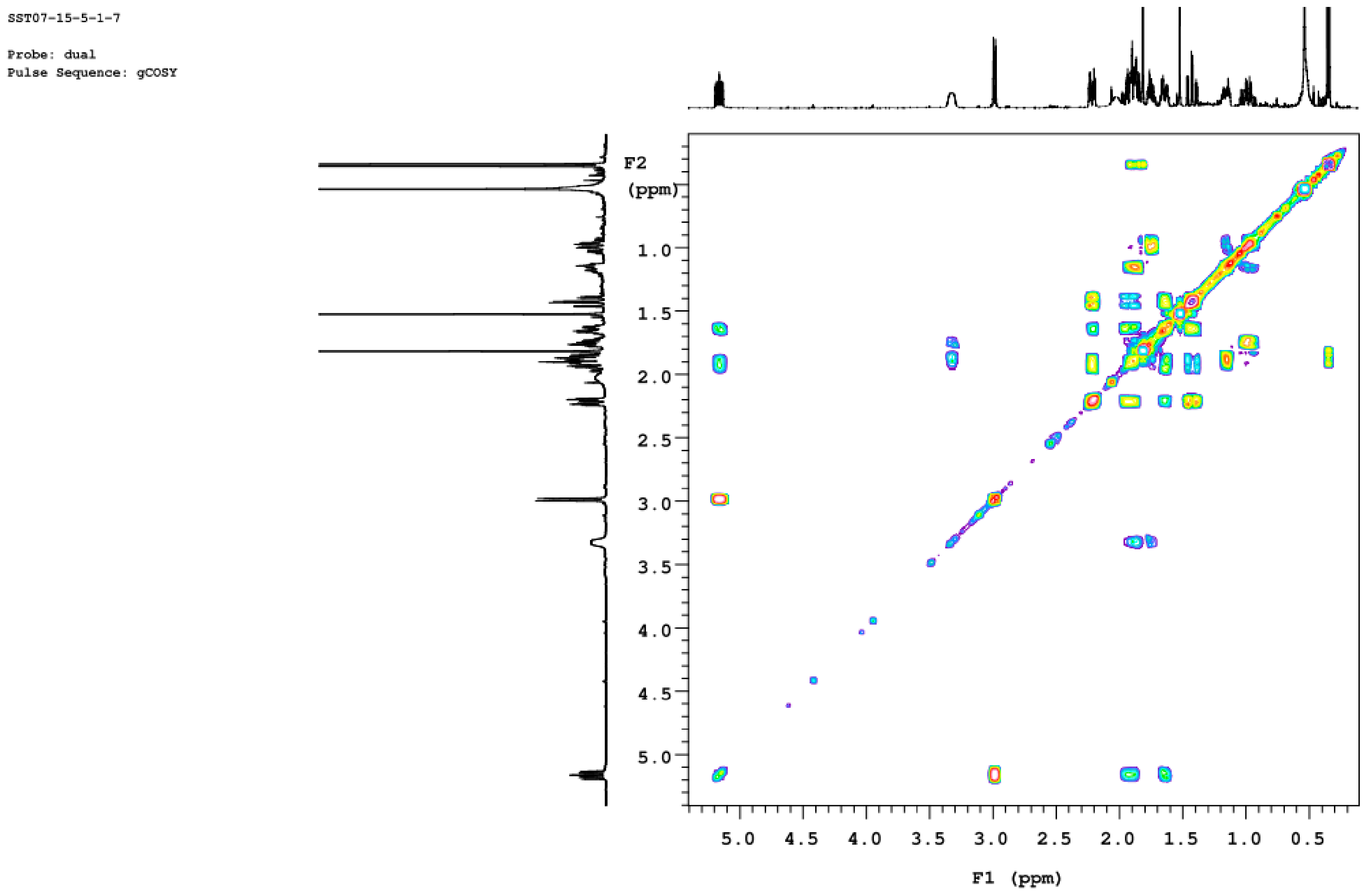

Figure S16. HSQC spectrum (400 MHz) of parathyrsoidin F (**3**) in C_6D_6.

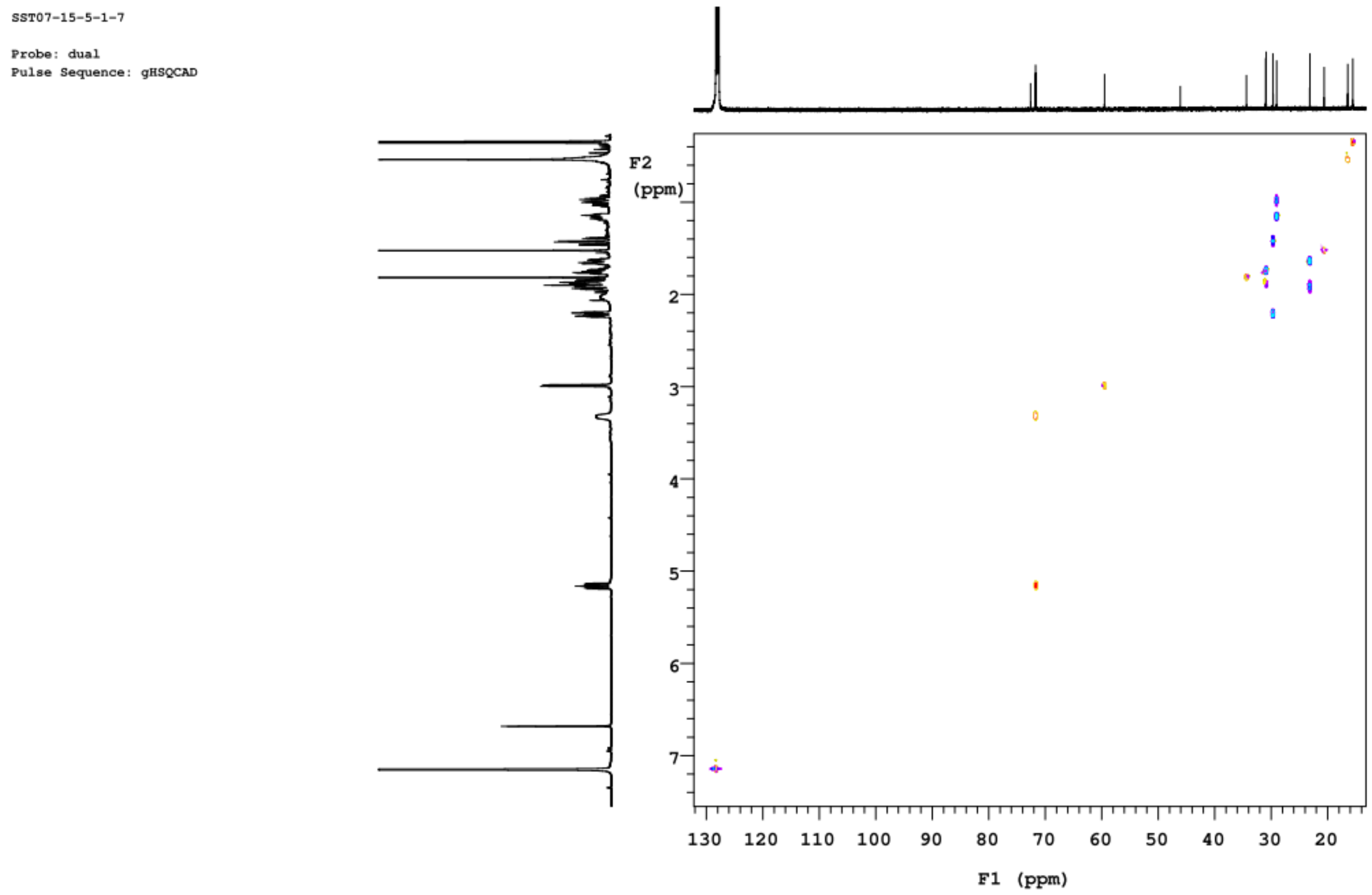

Figure S17. HMBC spectrum (400 MHz) of parathyrsoidin F (**3**) in C_6D_6.

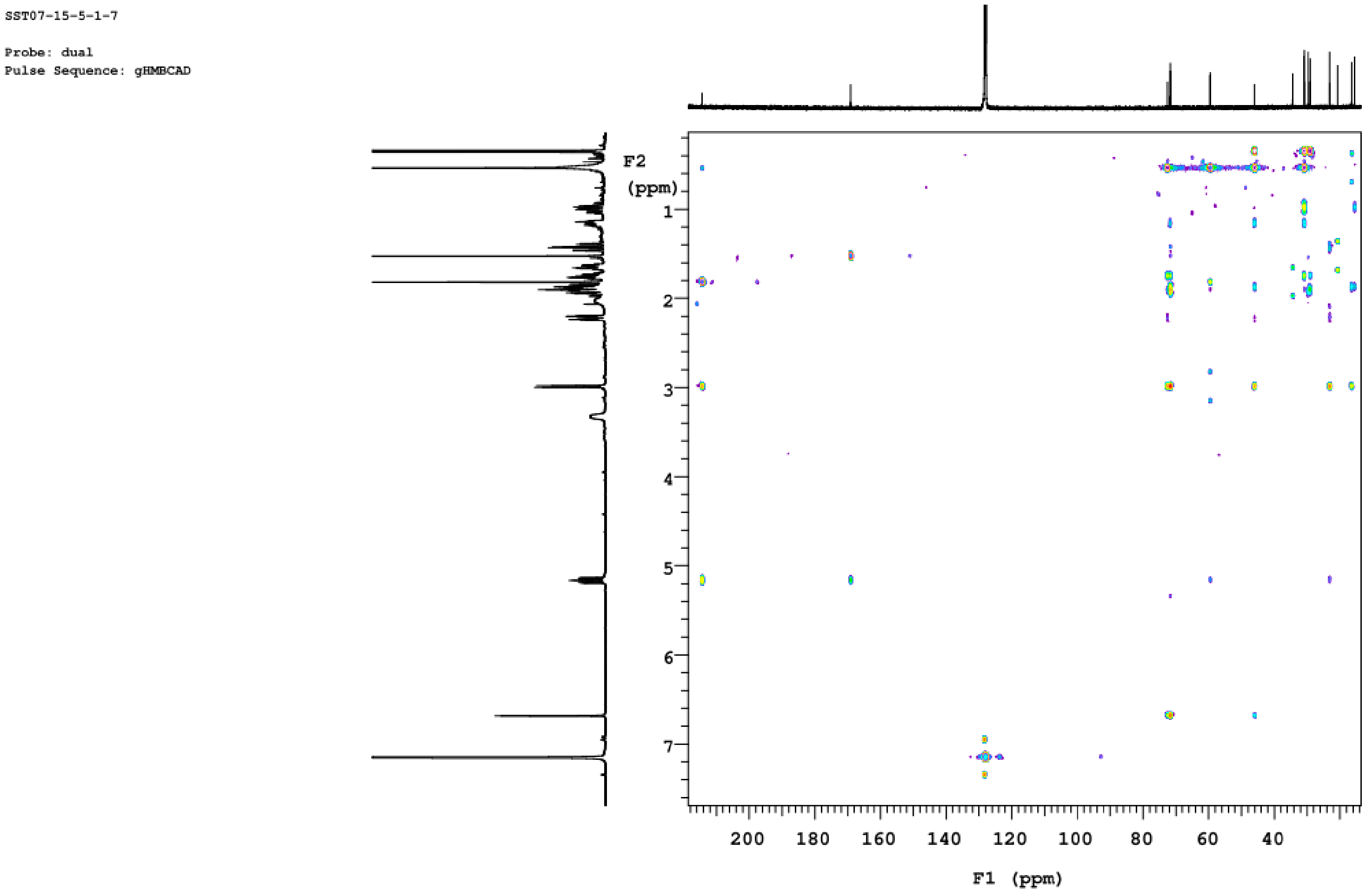

Figure S18. NOESY spectrum (400 MHz) of parathyrsoidin F (**3**) in C_6D_6.

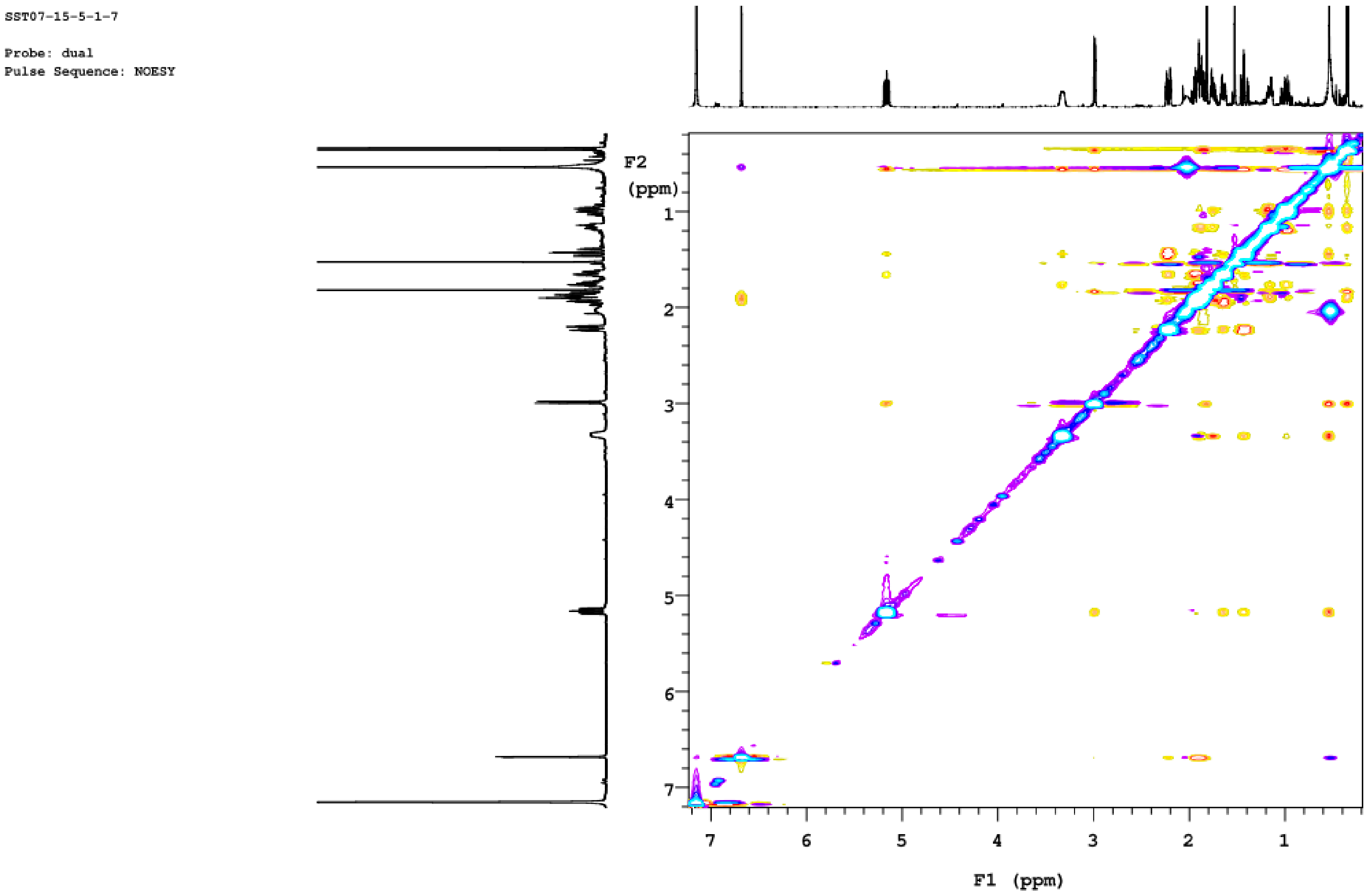

Figure S19. ^{1}H NMR spectrum (400 MHz) of parathyrsoidin G (**4**) in C_6D_6.

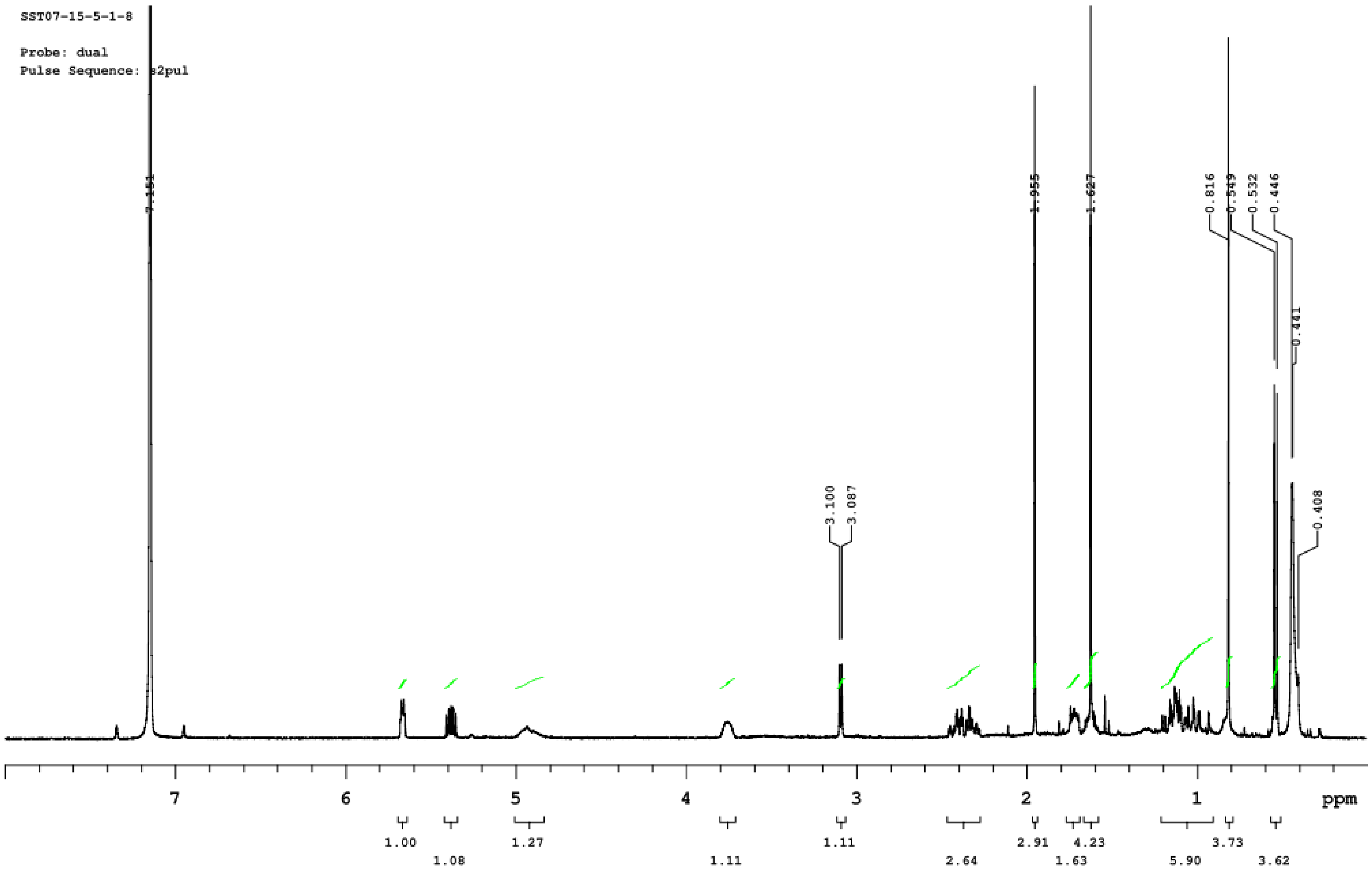

Figure S20. ^{13}C NMR spectrum (100 MHz) of parathyrsoidin G (**4**) in C_6D_6.

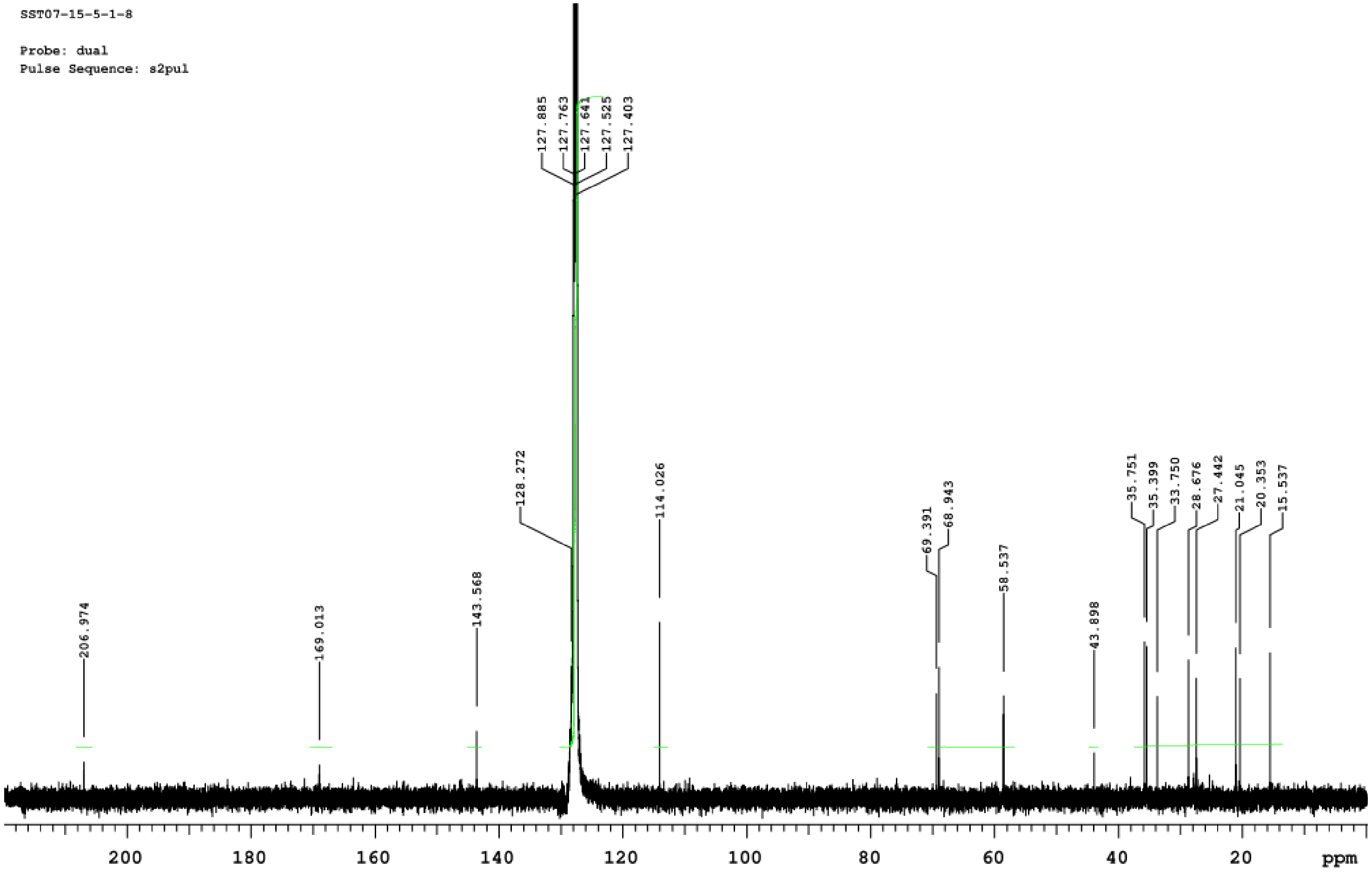

Figure S21. COSY spectrum (400 MHz) of parathyrsoidin G (**4)** in C_6D_6.

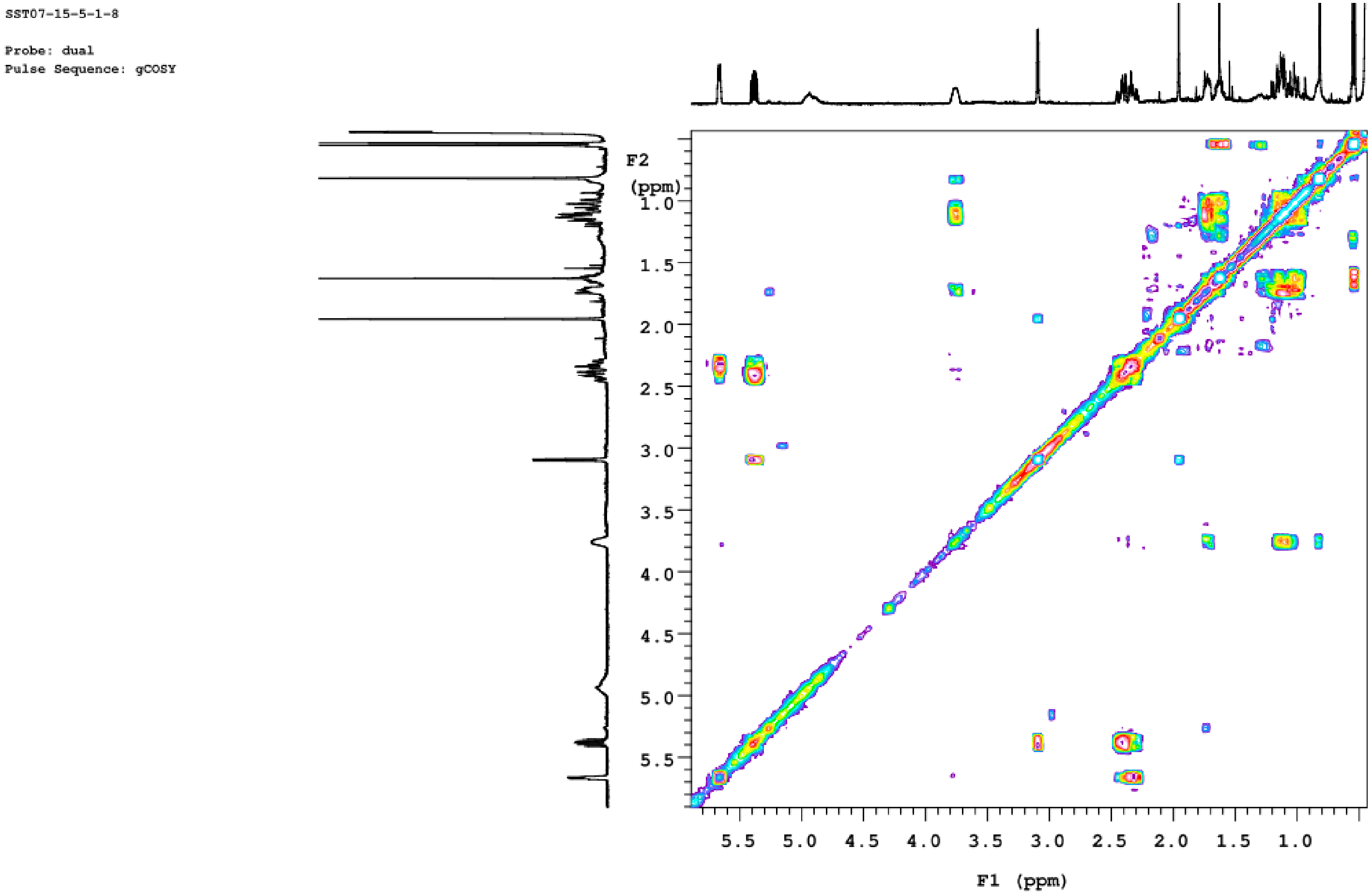

Figure S22. HSQC spectrum (400 MHz) of parathyrsoidin G (**4**) in C_6D_6.

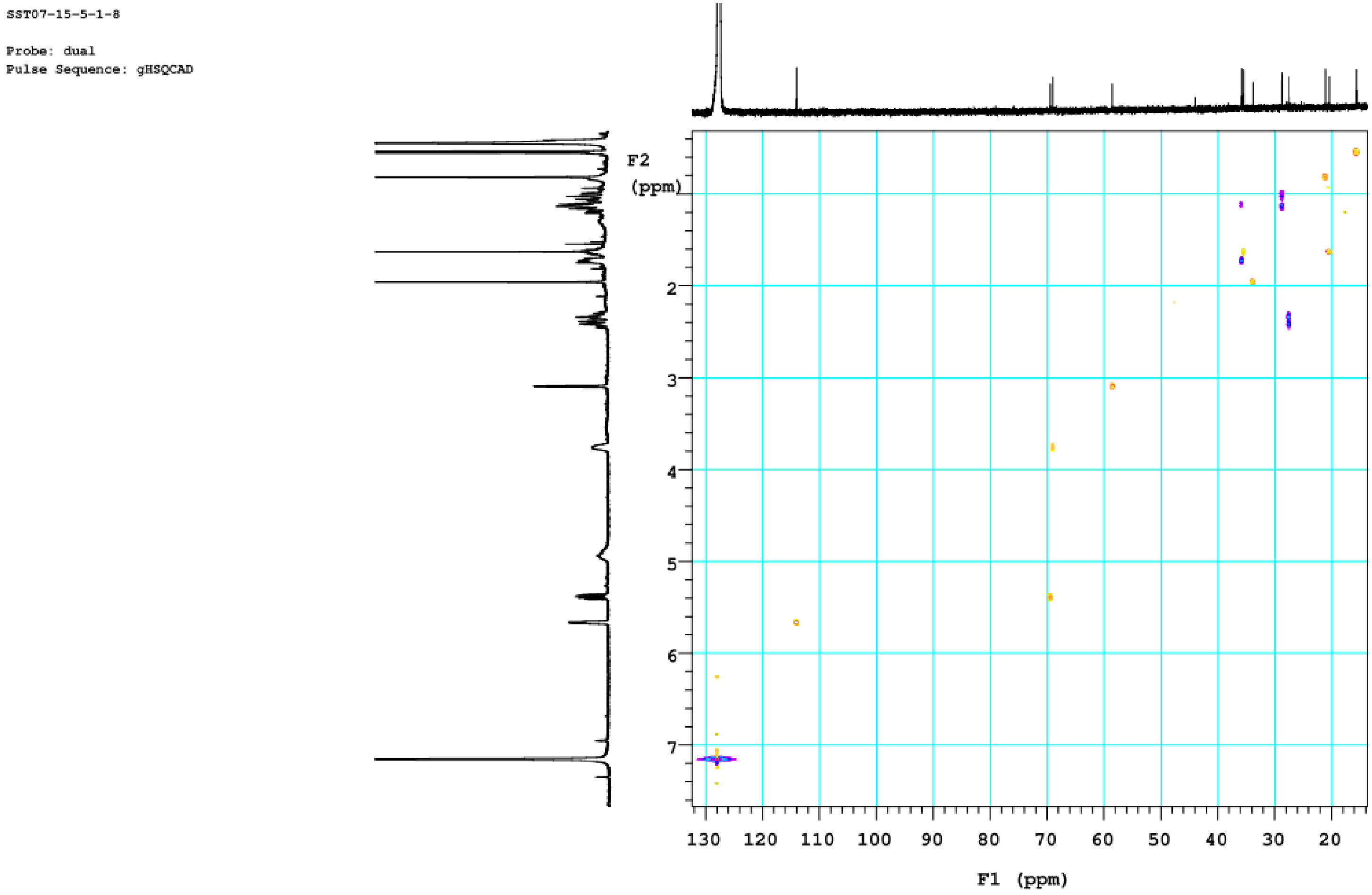

Figure S23. HMBC spectrum (400 MHz) of parathyrsoidin G (**4**) in C_6D_6.

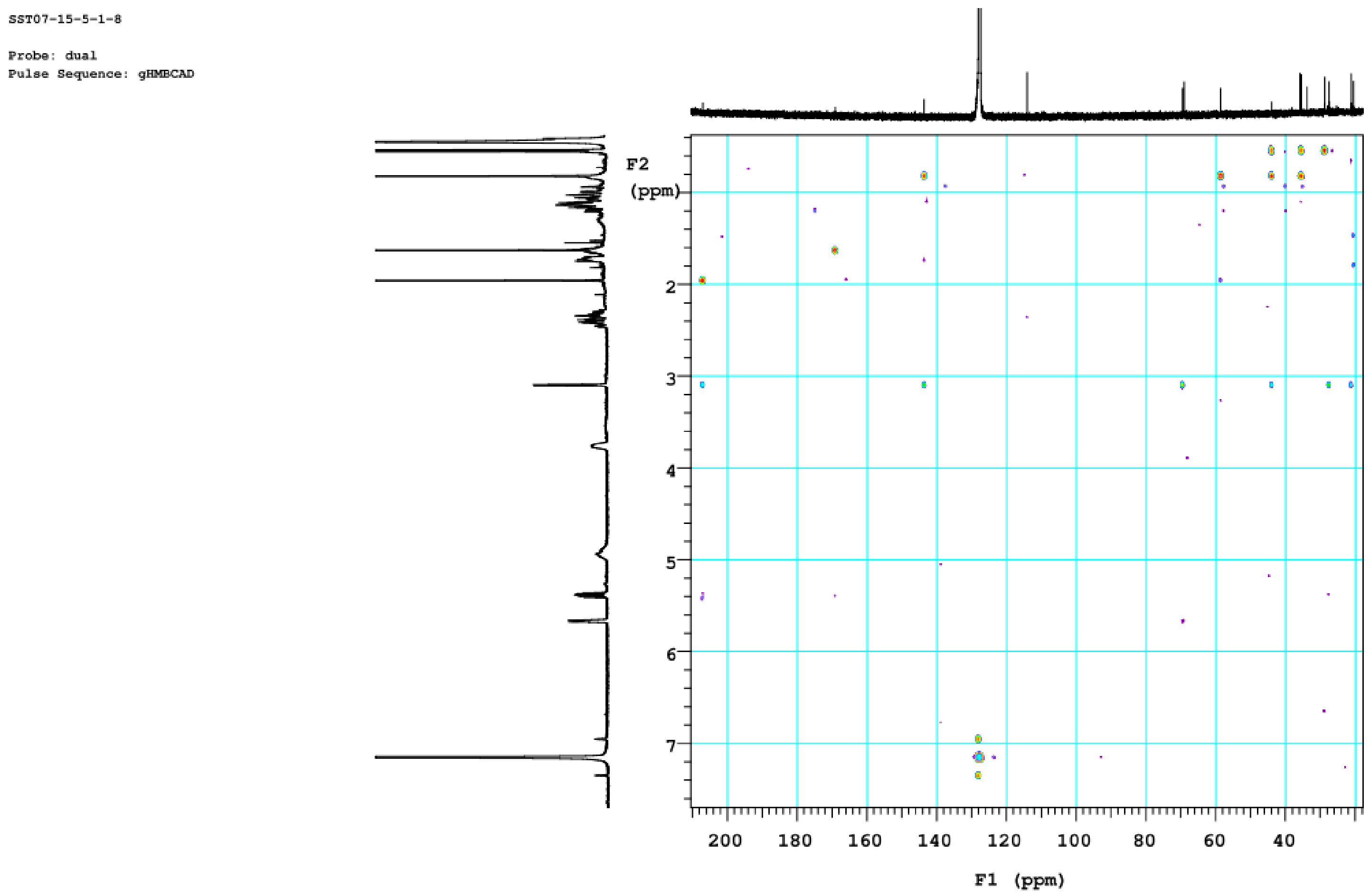

Figure S24. NOESY spectrum (400 MHz) of parathyrsoidin G (**4**) in C_6D_6.

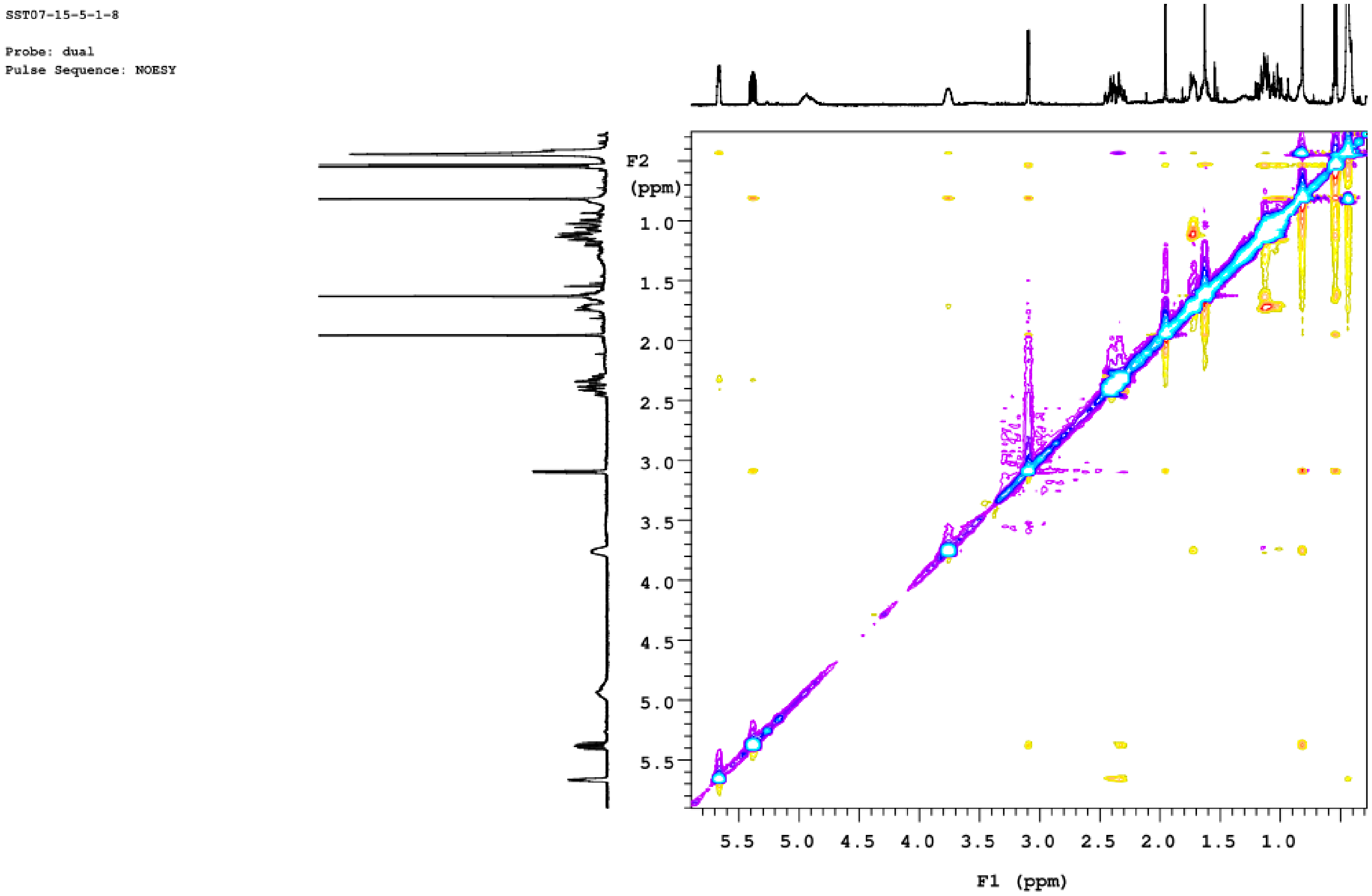

Supplement: Supplementary file 1 [file marinedrugs-15-00392-s001.pdf]
